# An impact evaluation of two rounds of mass drug administration on the prevalence of active trachoma: A clustered cross sectional survey

**DOI:** 10.1371/journal.pone.0201911

**Published:** 2018-08-29

**Authors:** Asrat Genet Amnie, Paul Emerson, Deborah McFarland, Jonathon King, Emmanuel Miri, Lisa Dickman

**Affiliations:** 1 Health Education Unit, Education Department, Eugenio María de Hostos Community College, The City University of New York, New York, NY, United States; 2 The Carter Center, Atlanta, GA, USA; 3 Rollins School of Public Health, Emory University, Atlanta, GA, USA; 4 The Carter Center, Jos, Plateau, Nigeria; London School of Hygiene and Tropical Medicine, UNITED KINGDOM

## Abstract

**Introduction:**

We investigated the impact of two round of mass drug administration on trachoma prevalence in Plateau and Nasarawa States of Nigeria. The mass drug administration was conducted as a component of the SAFE Strategy, a combination of interventions recommended for the global elimination of blinding trachoma.

**Methods:**

The study consisted of a two-stage cross-sectional clustered sample survey in which 3990 people from 793 households were screened for clinical signs of trachoma.

**Results:**

Of the total 3990 people examined, 1530 were children, of which 808 (53%) were boys and 704 (47%) were girls. The impact of intervention as measured by the changes in overall prevalence of follicular trachoma were as follows: At baseline the overall prevalence of follicular trachoma among children 1–9 years of age was 6.4%, 95% CI [5.8, 7.0]; the overall prevalence of trachomatous trichiasis in the total population was 0.20%, 95% CI [0.16, 0.25]. At follow up, the overall prevalence of follicular trachoma among children 1–9 years of age was 3.4%, 95% CI [1.9, 4.9]; the overall prevalence of trachomatous trichiasis in the total population was 0.20%, 95% CI [0.00, 0.05]. The highest statistically significant reduction (96%) in follicular trachoma prevalence was observed in Doma Local Government Area of Nasarawa State from baseline prevalence of 13.6%, 95% CI [9.7, 17.5] to follow-up prevalence of 0.5%, 95% CI [0.0, 1.5] and the lowest reduction (58%) in follicular trachoma prevalence was observed in Langtang North Local Government Area of Plateau State from baseline prevalence of 15.8%, 95% CI [9.3, 22.3] to 6.6%, 95% CI [1.6, 11.6], (p<0.05).

**Conclusion:**

A significant reduction in the overall prevalence of follicular trachoma was achieved after two rounds of mass drug administration. In the absence of significant activities pertaining to facial cleanliness and environmental sanitation components of the SAFE strategy in the intervention areas, the observed deep reductions in prevalence could mainly be attributed to mass drug administration. Therefore, two rounds of mass azithromycin administration may be as effective as guideline-recommended three or more rounds in reducing active trachoma prevalence but findings should be replicated in more robustly designed studies.

## Introduction

Trachoma is an endemic disease in over 50 countries worldwide, mostly in Africa and the Middle East. It is estimated that 40.6 million people suffer from active trachoma with 8.2 million estimated to have trichiasis, accounting for 3% of the global burden of blindness [1]. Blinding trachoma was targeted for elimination by the year 2020 through the SAFF Strategy. Elimination of trachoma as a public health problem is defined as: (i) a prevalence of trachomatous trichiasis of less than 0.2% in adults aged over 15 years (approximately 1 case per 1000 people); and (ii) a prevalence of less than 5% of trachomatous inflammation follicular in children aged 1–9 years [2].

SAFE combines elements of primary, secondary, and tertiary prevention: **S**urgical treatment for trachomatous trichiasis (tertiary prevention), **A**ntibiotic treatment for acute infection (secondary prevention), **F**acial cleanliness, and **E**nvironmental Sanitation (primary prevention) [2].

Among the African nations, Ethiopia has the highest known burden of active trachoma infection in the world [3]. Nigeria is also one of the countries endemic to neglected tropical diseases, including trachoma, onchocerciasis, and schistosomiasis [4, 5]. It is estimated that 1.13 million individuals aged 40 years or above are currently blind in Nigeria, with a further 3.1 million adults aged 40 years or above estimated to have moderate or severe visual impairment resulting from cataract, glaucoma, trachoma and other causes [6]. Nigeria like all other developing countries in the world has a number of public health challenges, including a disproportionately large concentration of the health workforce in urban tertiary health care services, and the presence of multiple government-regulated health care delivery systems: orthodox, alternative, and traditional, with varying degrees of access and levels of service delivery [7]. Nigeria is one of the countries which actively implemented the WHO-endorsed SAFE strategy for trachoma control and elimination. Other African countries, actively implementing the SAFE strategy include Ethiopia, Mali, Sudan, South Sudan, and Uganda. The Nigeria Trachoma Control program is a component of a collaborative efforts of the Carter Center and Nigeria's Federal Ministry of Health launched to address widespread tropical infectious diseases such as Guinea worm, lymphatic filariasis, schistosomiasis, river blindness, malaria, as well as trachoma [8]. Trachoma is a disease of entire communities in sub-Saharan Africa. The pool of infection resides predominantly in children but the blinding effect impacts mainly the adult population. The WHO classifies Chlamydial trachomatis infection of the conjunctivae into five categories: Follicular Trachoma (TF), Inflammatory Trachoma (TI), Trachomatous scarring (TS), Trachomatous Trichiasis (TT), and Corneal Opacity (CO). Communities with TF prevalence above 5% require mass antibiotic treatment. As per the guidelines, mass treatment with antibiotics annually for at least 3–5 years is carried out in trachoma-endemic communities. Standard WHO guidelines recommend three rounds of antibiotic treatment as part of the SAFE Strategy [9].

Trachoma control programs like any other public health intervention efforts for Neglected Tropical Disease have tremendous resource constraints. Research evidence from SAFE Strategy implementation indicates that an enhanced implementation of the prevention-focused components of the SAFE strategy would minimize a build-up in the backlog of trachomatous trichiasis cases who need surgery but would also require sustained advocacy efforts that would lead policymakers and program practitioners to adopt novel intervention approaches with sustained community engagement for trachoma elimination [10–13]. Public health advocacy efforts have the power to mobilize the community, involve stakeholders, and communicate to policy-makers to take measures to address priority public health problems. This study is an interim impact evaluation of two rounds of Mass Drug Administration (MDA) as a component of the SAFE strategy for trachoma control program in two high-prevalence trachoma endemic States in Nigeria.

### Rationale and objectives of the study

Monitoring and evaluation should be an integral component of public health program implementation. This interim evaluation of the trachoma control intervention in Nasarawa and Plateau States of Nigeria was conducted in order to document the level of success, or lack thereof, in achieving the objectives of the MDA; to identify areas of the program that need improvement; to decide how to allocate resources; to mobilize community support; to redistribute or expand the locations where the intervention is carried out; to improve the content of the program’s health promotion materials; to focus program resources on a specific population and possibly to plan more funds for additional activities. The primary outcome of the study was to determine if there was a statistically significant difference in the prevalence of TF in children ages 1 to 9 years compared to baseline TF prevalence in the same age group after two rounds of MDA. The objectives of the study included: (1) to undertake an assessment of the impact of the Antibiotic Treatment component of the SAFE Strategy, i.e., mass drug administration on trachoma prevalence by determining the level of reduction in the prevalence of trachomatous follicular inflammation from baseline levels after two rounds of mass drug administration, (2) to identify the impact of the SAFE Strategy trachoma control program interventions on latrine ownership and use and antibiotic coverage, and (3) to recommend areas of improvement for future program intervention.

## Materials and methods

### Study area

The intervention areas were three Local Government Areas (LGAs) of the Plateau State and four LGAs of Nasarawa State of Nigeria. This survey included all the three LGAs in Plateau State and four LGAs in Nasarawa State, located in the Central Region of the country [S2 Table]. After baseline trachoma–prevalence survey was conducted in 2008/2009 [14], mass drug administration (MDA) with azithromycin for trachoma control was conducted in 2010 and 2011 in all the trachoma endemic areas of Nasarawa and Plateau States which had baseline trachoma prevalence of 10% or above. The evaluation survey was carried out during the month of June 2012.

### Study design, study population, and sampling method

A two-stage cluster sampling was used to determine the sample from study population in Seven LGAs of the States of Plateau and Nasarawa. In the first stage, 40 clusters were identified by systematic random sampling. The survey team visited 793 households selected from identified segments of the 40 clusters with each cluster contributing the number of households proportional to their population size [S3 Table]. In each household, informed consent was obtained from the head of the household (or his or her adult representative) before the interviews began [S1 File].

In the context of this study, a household consists not only of the basic family unit of parents and their children but extends to include other adults and children living under the same roof. In cases where there have been second (and more) marriages with or without children, this is considered one household, regardless of whether the individuals live together in a single household or separately if they live in the same compound. Thus, a household includes all the individuals who occupy a housing unit as their usual place of residence.

### Data collection, data management, and analysis

First, training of data collectors was conducted. Training was provided to community trachoma graders and data collectors by qualified international and local professionals in Nigeria from June 5 to June 9, 2012[S4 Table]. The recruitment of trachoma graders and data collectors was conducted as per criteria established by local health authorities of Nigeria. The training course included the purpose of the survey; protocol roles and responsibilities; trachoma overview and the SAFE strategy; basic, clinical and epidemiologic concepts such as anatomy of the eye, examination techniques and trachoma grading based on WHO clinical criteria; sampling including mapping, segmenting, data collection of both household characteristics and individual census through field exercise in Langtang South School Children; introduction to the use of the electronic device, the tablet, for data collection through role play. A standard pre-coded questionnaire was administered to each head of household to collect basic household demographic data and knowledge of trachoma disease, the trachoma prevention strategy called SAFE (short for Surgery, Antibiotics, Facial cleanliness, and Environmental sanitation) and the household-level interaction with the community directed distributor (CDD) during mass drug administration [S1 Table]. It is to be noted that community-directed distributors (CDDs) are the men and women volunteers selected by their communities and trained for the annual distribution of drugs in MDA campaigns: Zithromax for follicular trachoma, ivermectin for onchocerciasis, ivermectin-albendazole for lymphatic filariasis, and praziquantel for schistosomiasis [15]. In Nigeria, NTD-interventions, including antibiotic distribution are often integrated to enhance program efficiency, unlike in many other countries where MDA is usually conducted by salaried government-employee health workers.

The survey teams also documented the physical presence or absence of a household pit latrine and a visual inspection looking for evidence of the use of the latrine by the household was made if they own a latrine. After the head of a household interview was completed, the members of the entire household were enumerated on a census form. Each member was asked to report their age, type of antibiotic taken (if any), and reason for not participating, if applicable. Children ages 1–9 years of age were clinically examined for trachoma using x2.5 binocular under adequate light. Each eye was graded using the WHO standard and multiple grades of trachoma were recorded for each eye depending on the type of lesion identified by the clinical examination if present. Individuals were shown Zithromax (Pfizer-supplied drug used to treat active trachoma) bottles and the pills to avoid confusion with other mass drug administration (MDA) programs. Survey data were entered in electronic tablets at the time of data collection and validated using Microsoft Excel 2007 [16]. The analysis was conducted using SAS version 9.3 (The SAS Institute Cary, NC) [17]. The percentage reduction in the prevalence of the outcomes was calculated, and comparison of baseline and follow-up proportions was made using 95% Confidence Intervals. The CHISQ is the test of significance in the SURVEYFREQ procedure. The difference between baseline and follow up prevalence or coverage was taken as statistically significant if p<0.5% and/or the confidence intervals were not overlapping.

### Ethical considerations

The study received approval from the Institutional Review Board of Emory University under Paper Study Number 079–2006 and from the Ministry of Health of Nigeria. Standardized consent statements were read in the local languages to the village head by trachoma survey teams upon their arrival to the community, to request permission to enter the community. Verbal informed consent was obtained from the head of the household prior to conducting interviews [S1 File]. No incentives were offered or provided to any participant. Strict confidentiality and anonymity of study participants was maintained during data analysis.

## Results

### Sociodemographic characteristics

In this follow up survey, the majority of heads of households 79% (625 out of 793) were women. Thirty-four percent of the respondents in Nasarawa and 29 percent in Plateau did not go to any formal or religious school. Sixty-nine percent of the respondents in Nasarawa and 60% in Plateau state were farmers or cattle herders. In this survey, 3990 people from 793 households were examined out of which 38% (1530) were children. Of the 1530 children, 808 (53%) boys and 704 (47%) girls, aged l-9 years were screened for clinical signs of trachoma. A total of 2138 persons, 1014 (46%) males, and 1124 (54%) females, above the age of 14 years were also examined for signs of trachoma.

## Trachoma prevalence

**Fig 1:**
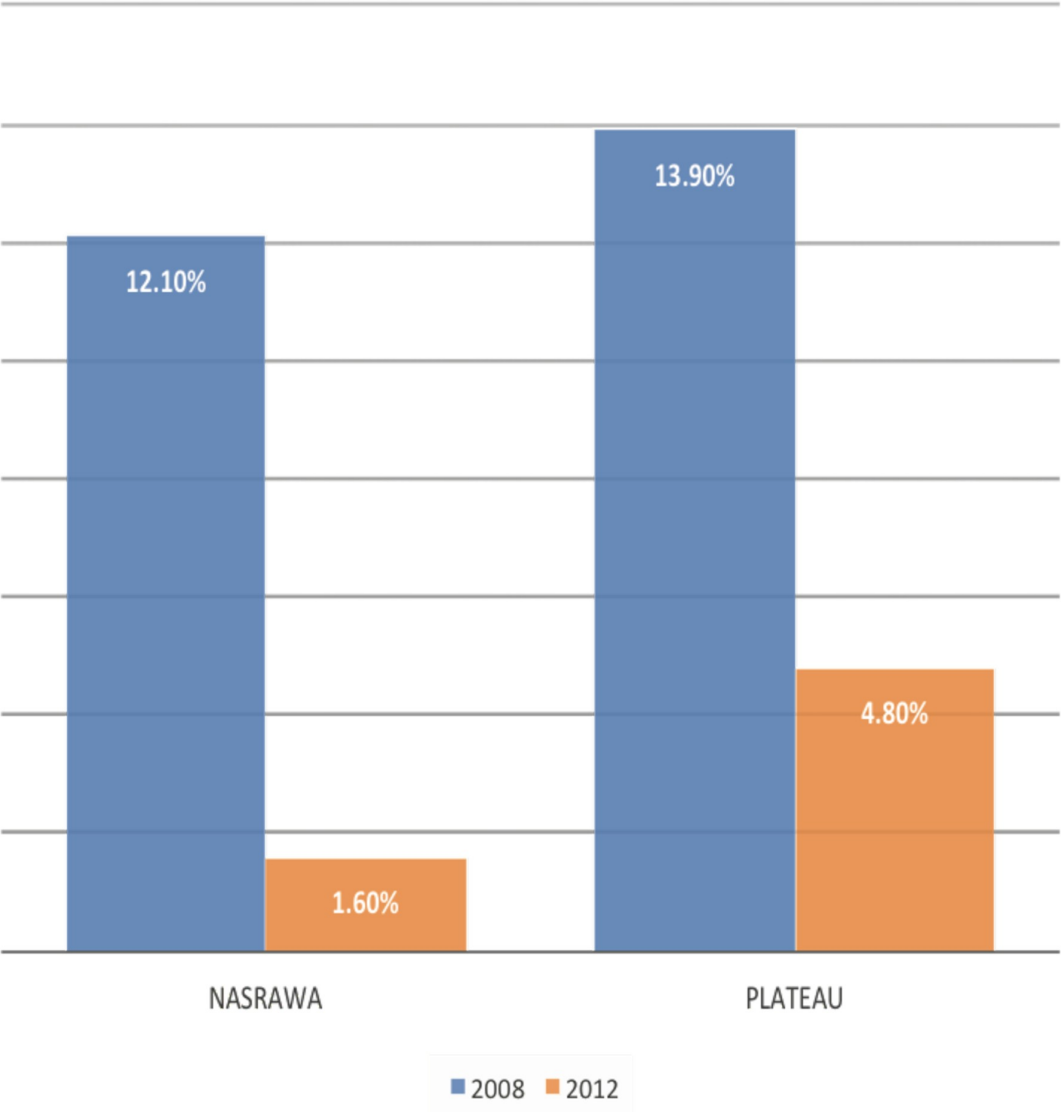
Follicular trachoma TF prevalence in Nasarawa and Plateau States, 2008/2012.

**Fig 2:**
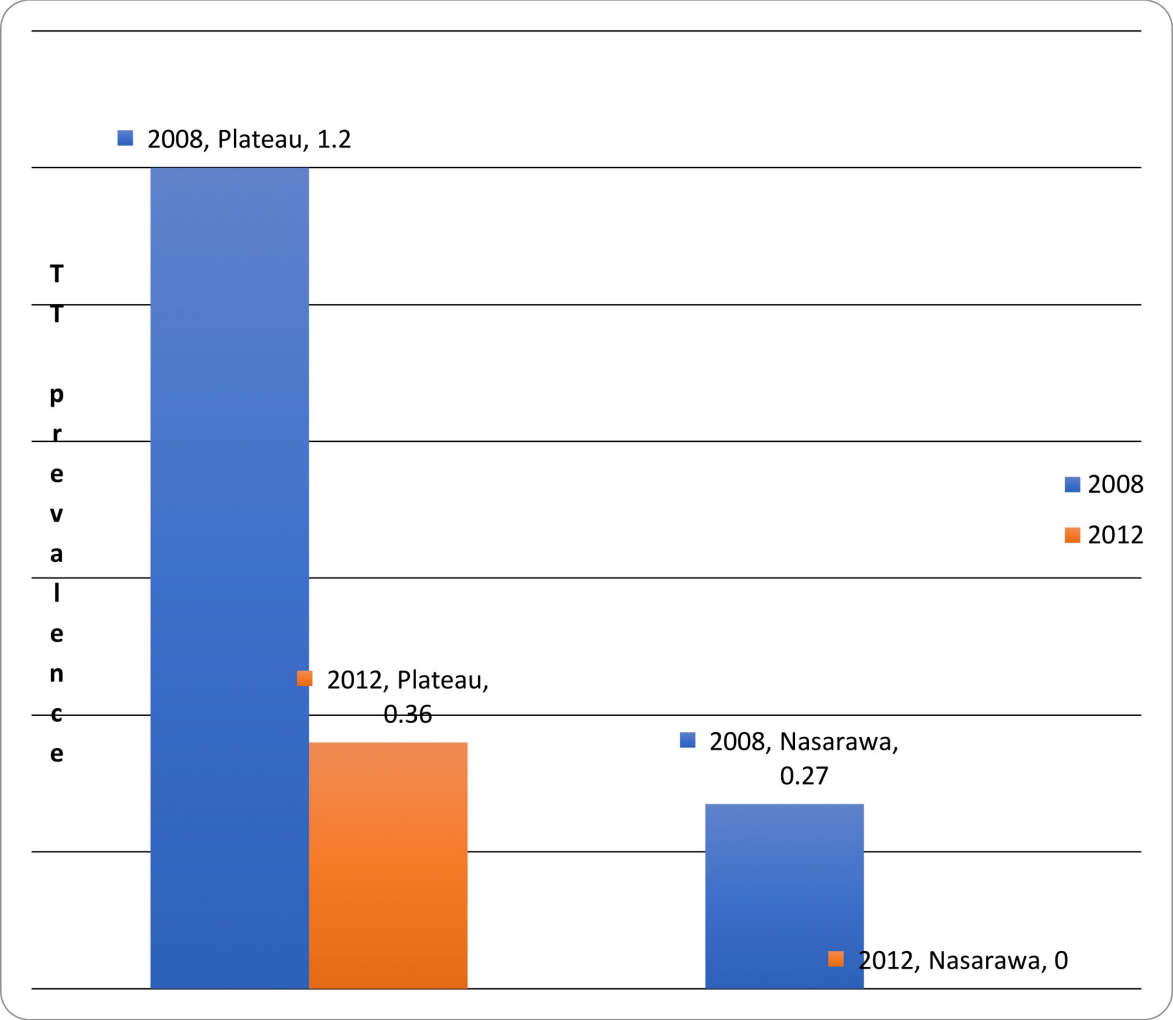
Trachomatous trichiasis TT Prevalence in Nasarawa and Plateau States, 2008/2012.

The impact of intervention as measured by the changes in the prevalence of Follicular Trachoma (TF) were as follows: at baseline the overall prevalence of TF among children 1–9 years of age was 6.4%, 95% CI [5.8, 7.0]; the overall prevalence of TT in the total population was 0.20%, 95% CI [0.16, 0.25]. About two years later at follow up, the overall prevalence of TF among children 1–9 years of age was 3.4%, 95% CI [1.9, 4.9]; the overall prevalence of TT in the total population was 0.20%, 95% CI [0.00, 0.05%]. The highest statistically significant reduction (96%) in TF prevalence was observed in Doma LGA of Nasarawa State from baseline prevalence of 13.6%, 95% CI [9.7, 17.5] to follow-up prevalence of 0.5%, 95% CI [0.0, 1.5] and the lowest reduction (58%) in TF prevalence was observed in Langtang North LGA of Plateau State from baseline prevalence of 15.85, 95% CI [9.3, 22.3] to 6.6%, 95% CI [1.6, 11.6] which was also statistically insignificant (p<0.05). The state-level findings are displayed in Figs 1 and 2.

**Fig 3:**
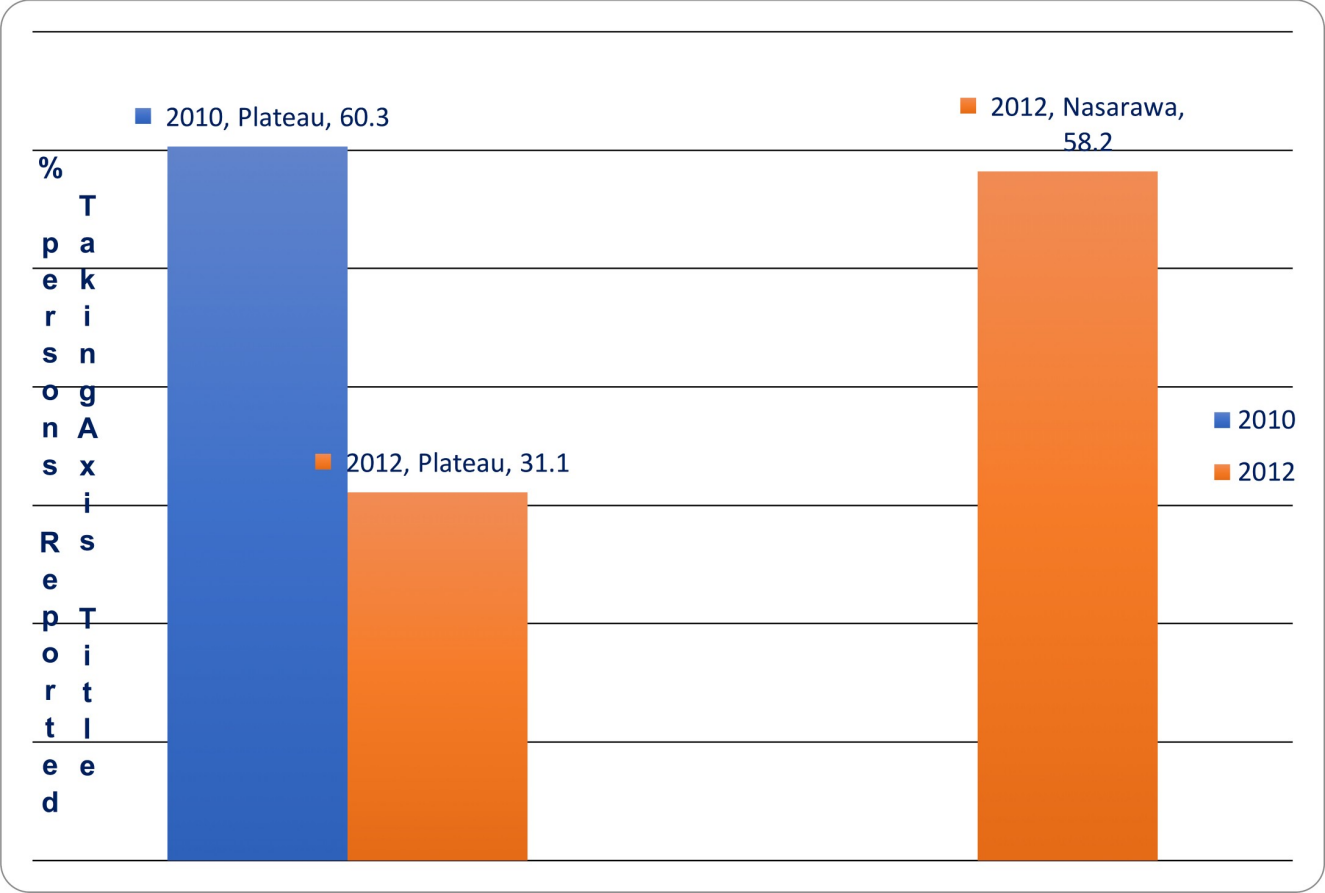
MDA coverage at baseline and follow up surveys in the two States of Nigeria, 2010, 2012.

Antibiotic coverage was 60.3% for Plateau State at baseline and 31.1% at follow up surveys respectively. The follow up antibiotic coverage for Nasarawa was 58.2%. Baseline survey did not capture antibiotic coverage in Nasarawa state despite the conduct of MDA in that State in 2010 (Fig 3).

### Environmental sanitation

**Fig 4:**
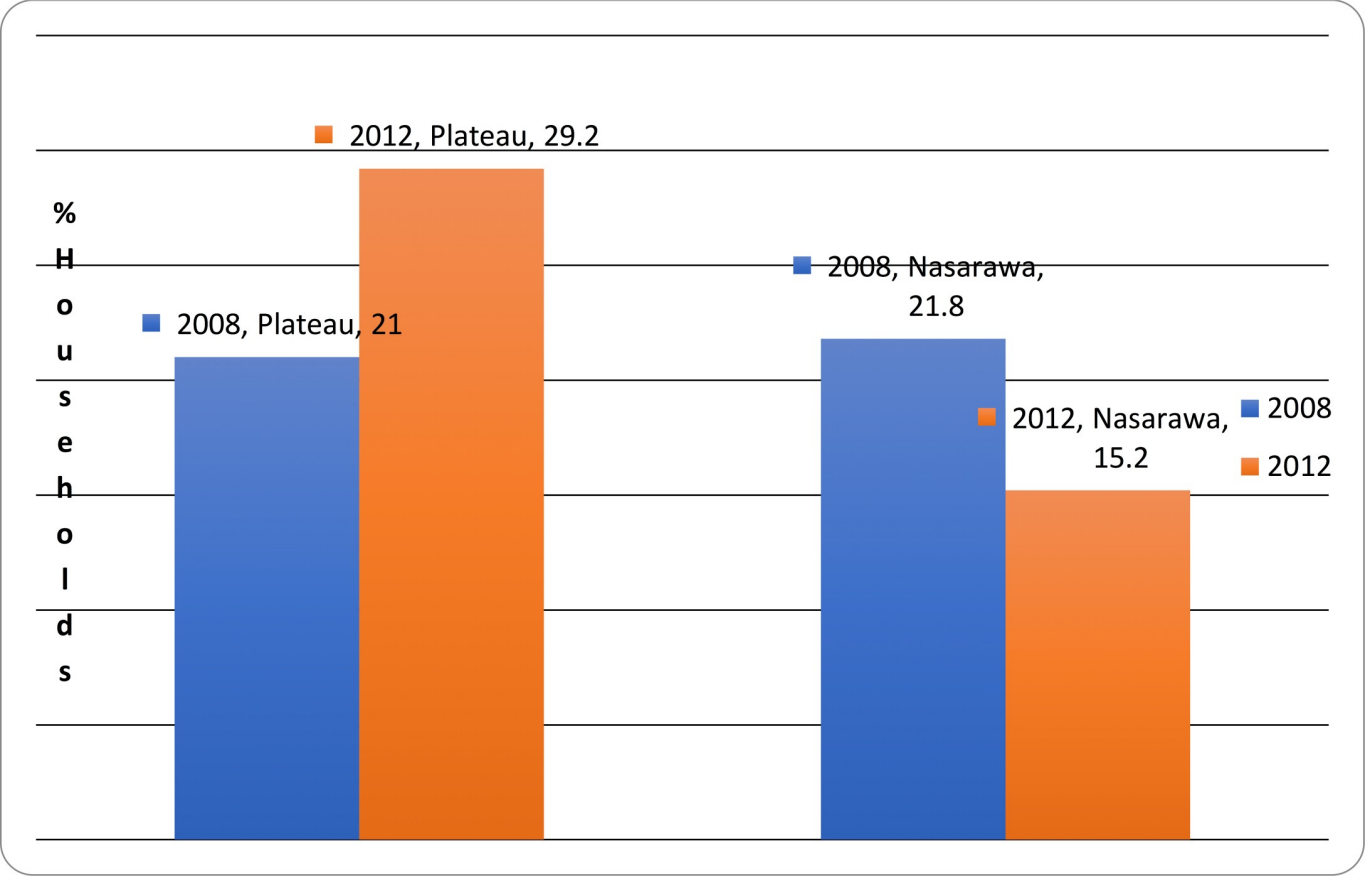
Household latrine ownership in Plateau and Nasarawa States of Nigeria 2008/2012.

Household latrine coverage was 21.0% and 29.2% for Plateau State and 21.8% and 15.2% for Nasarawa State at baseline and follow up surveys respectively (Fig 4). Latrine Use at baseline survey was 100% for Nasarawa State and 97.5% for Plateau State At follow up survey, latrine use was 100% for Nasarawa State and 98.2% for Plateau State.

## Discussion

Mass drug administration for control of trachoma was carried out in Nasarawa and Plateau states of Nigeria in the years 2010 and 2011 based on the findings of the baseline surveys conducted during the years 2008 and 2009 [14]. The prevalence of follicular trachoma at base line which was high enough to justify the introduction of mass treatment was reduced statistically significantly following two rounds of MDA in the absence significant activities for the implementation of facial cleanliness and environmental sanitation components of the SAFE strategy. In a similar study [18], the prevalence of trachoma inflammation-follicular (TF) dropped from 36.7%, 95% CI [33.9, 39.6] to 18.4%, 95% CI [15.4, 21.8], an overall prevalence reduction of 49.9% after three years of intervention. This is compared to a drop from 6.4%, 95% CI [5.8, 7.0] to 3.4%, 95% CI [1.9, 4.9] which is a 47% prevalence reduction for the seven LGAs after two years of intervention in our study. Another study [19] reported 32% reduction in TF and 45% drop in TT in Amhara Region of Ethiopia where the SAFE strategy was enhanced through bi-annual mass-mobilization campaigns. In our study, the reduction in the prevalence of trachomatous trichiasis (TT) for the seven LGAs from 0.69%, 95% CI [0.48, 0.90] to 0.21%, 95% CI [0.0, 0.47] was statistically significant. This finding is similar to findings by a study on the effects of intervention with the SAFE strategy on trachoma where there was a significant reduction in prevalence with antibiotic (TF), face washing (clean face) and environmental (latrine) components [20]. Antibiotic distribution to a household is strongly associated with reduction in active trachoma in our findings consistent with findings in other studies [21]. Antibiotic coverage was 60.3% and 31.1% for plateau State at baseline and follow up surveys respectively. The follow up antibiotic coverage for Nasarawa was 58.2%. Baseline survey did not capture antibiotic coverage for Nasarawa state despite the conduct of MDA in that State in 2010 [17]. These antibiotic coverage rates are significantly lower compared to the WHO ultimate intervention goal of at least 80% coverage. Study results from multiple clinical trials indicate that antibiotic treatment reduced the prevalence of active trachoma and ocular infection one year after single-dose treatment and oral azithromycin was more effective than topical tetracycline [22]. The relative contribution of other components of the SAFE strategy could not be estimated in this study. However, a study in Ethiopia on active trachoma and community sanitation indicated that there was no clear evidence of the effect of community sanitation usage on active trachoma by household latrine use and water access [23], perhaps due to the effect of neighborhood factors on the overall status and effect of environmental sanitation. In a similar study, surgery had no impact on the prevalence of trachomatous trichiasis [18]. A review of clinical trials indicates that two trials on latrine provision as a fly control measure have not demonstrated significant trachoma reduction [24].

There have not been any major socio-demographic events during the intervention period in both Plateau and Nasarawa states such as internal displacement of people or immigration of populations from neighboring states or countries. This is important because changes in public health status could be reasonably attributed to the effect of the intervention.

The remarkably low latrine coverage (baseline and follow-up rate of 21.0% and 29.2% for the Plateau and 21.8% and 15.2% for Nasarawa respectively) were associated partly with the temporary nature of latrine infrastructure. A similar study indicates that only about 30% of the Nigerian populace has access to the proper sanitary system, and in rural areas, this percentage drops to less than 25% [25]. The lack of significant improvement in latrine ownership in Plateau state and the decline in ownership observed in Nasrawa state are indicative of the need for strengthening the environmental component of the SAFE Strategy in the intervention areas.

## Limitations of the study

As a limitation to the study, the inherent weaknesses of cross-sectional designs limit the ability to establish a total attribution of the impact to the intervention. Another limitation is the inability to rule out the existence of confounding and counterfactual non- program factors which could significantly alter the outcome of the study. An important limitation of this study includes the inability measure the relative contribution of each component of the SAFE Strategy because of lack of activity and data on facial cleanliness and environmental sanitation components of the program. Research using prospective longitudinal designs which employ control groups could provide better evidence in terms of how much of the outcome could be attributed to the intervention but would require a longer time and higher costs. Understanding the individual impact of surgery, facial cleanliness and environmental sanitation interventions would require a full scale SAFE Strategy implementation and impact evaluation.

## Conclusion

The findings of this study indicate that two rounds of MDA may be as effective as three or more rounds of MDA in reducing TF prevalence. However, findings need to be replicated in more robust designs before programmatic and policy recommendations could be made to practitioners and policymakers. A significant reduction in the prevalence of TF was registered after two rounds of MDA in the two States of Nigeria. Our finding of post-intervention TF prevalence was below 5% in the two States. Thus, two rounds of MDA may be as effective as three or more rounds of WHO-guideline recommendations in reducing TF prevalence. However, prevention of re-introduction from outside to treated communities is important to ensure sustainability. Moreover, implementation of the full package of the SAFE strategy could further reduce the need for more than two rounds of antibiotics. If these findings are reproducible, resources used for conducting the third and subsequent rounds of MDA could be shifted to hard-to-reach communities and other areas in dire need of a trachoma control program. The coverage with MDA has been consistently below the 80%, yet significant reductions in TF have been observed which is indicative of the need to enhance close monitoring and strengthen the distribution mechanism to ensure adequate coverage in the future. Capacity strengthening should ensure local ownership and sustainability of operations for trachoma control programs with an equal focus on environmental measures and assist the state governments of Nigeria and partners to achieve the ultimate intervention goals and the eventual goal of eliminating blinding trachoma through enhanced implementation of the SAFE strategy.

## Supporting information

S1 File Consent, procedure, and confidentiality information for adult study participants. This is a statement of Informed Consent, outlining the procedure, benefits, risks, participant rights, confidentiality and voluntary participation in and withdrawal from the study.
(DOC)

S2 File Checklist of recommendations done in the study as per STROBE statement. Checklist of items that were included in reports of *cross-sectional studies*
(DOC)

S3 File STROBE statement—Checklist of items that should be included in reports of *cross-sectional studies*. (DOC)

S1 Table Questionnaire for Nigeria MDA coverage Survey, June 2012. The questionnaire was designed for mass drug administration coverage Survey and has the following sections: (1) Respondents Demographics (2) Household Possessions (HP); (3) Knowledge of Mass Drug Administration of participants (M); (4) Questions on Water Supply and Sanitation (WS); (5) Use of Mosquito Nets (MN); (6) Household Socioeconomic Status (ES); (7) Possession of Latrine (PL); (8) A chart to collect the results of eye examination by a trained eye care professional. The questions have been coded RD1, RD2, RD3, etc. to denote Respondents’ Demographics; HP3, HP4, HP5, to denote house Hold Possession. As this questionnaire is a subset of a larger questionnaire used in trachoma surveys, questions not relevant for this study were omitted (for example, HP1, and HP2). The questionnaire also consists of information not relevant for this manuscript such as the use of mosquito bed nets for malaria prevention. In the program areas, malaria prevention and trachoma prevention are jointly implemented. Information is saved into electronic tablets by field workers as they interview heads of the households and household members.
(DOC)

S2 Table List of LGAs and enumeration areas of the study area and assigned cluster numbers. This consists of information on the Local Government Areas (LGA) of the two states of Plateau and Nasarawa. Each LGA is divided into several Enumeration Areas (used for census purposes in Nigeria) which are assigned cluster numbers.
(DOC)

S3 Table Clusters selected for study by systematic random sampling in each enumeration area. A total of 40 enumeration areas were selected by systematic random sampling. Each enumeration area is assigned a cluster number, and is divided into a weighted number of segments based on population density. Each enumeration area. is noted if it has a CDD (a Community Directed Drug Distributor assigned). Each segment may have as many as 15–20 household selected for study.
(DOC)

S4 Table Training agenda: Trachoma impact evaluation survey, plateau and nasarawa States, June, 2012. This is the schedule of a 5-day training of trachoma graders, and the use of electronic tablets for data collection.
(DOC)
