# Correction: An impact evaluation of two rounds of mass drug administration on the prevalence of active trachoma: A clustered cross sectional survey

**DOI:** 10.1371/journal.pone.0222660

**Published:** 2019-09-16

**Authors:** Asrat Genet Amnie, Paul Emerson, Deborah McFarland, Jonathon King, Emmanuel Miri, Lisa Dickman

## Notice of republication

This article was republished on September 3, 2019 to correct for errors in the byline. Paul Emerson, Deborah McFarland, Jonathon King, Emmanuel Miri, and Lisa Dickman should be included in the author byline instead of the Acknowledgments. Please see the correct author order, affiliations, contributions, and citation here:

Asrat Genet Amnie^1^, Paul Emerson^2^, Deborah McFarland^3^, Jonathon King^2^, Emmanuel Miri^4^, and Lisa Dickman^2^

**1** Health Education Unit, Education Department, Eugenio María de Hostos Community College, The City University of New York, New York, NY, United States **2** The Carter Center, Atlanta, GA, USA **3** Rollins School of Public Health, Emory University, Atlanta, GA, USA **4** The Carter Center, Jos, Plateau, Nigeria.

Conceptualization: AGA PE EM. Data Curation: AGA LD. Formal Analysis: AGA. Funding Acquisition: AGA PE. Investigation: AGA EM, LD. Methodology: AGA JK. Project Administration: AGA PE. Resources: AGA PE. Software: AGA JK. Supervision: AGA DM. Validation: AGA. Visualization: AGA. Writing–Original Draft Preparation: AGA. Writing- Review & Editing: AGA.

Amnie AG, Emerson P, McFarland D, King J, Miri E, Dickman L (2018) An impact evaluation of two rounds of mass drug administration on the prevalence of active trachoma: A clustered cross sectional survey. PLoS ONE 13(8): e0201911. https://doi.org/10.1371/journal.pone.0201911

The article’s Acknowledgments section has been updated to reflect this change. The correct Acknowledgments is as follows: I also want to acknowledge Dr. Nimzing F Jip, Dr. Egege and the entire Nigerian Carter Centre field professionals and staff for excellent team work.

Additionally, an incorrect version of Fig 1, without the correct follicular trachoma prevalence values, was published in error. The correct follicular trachoma prevalence for Nasarawa State in 2012 was 1.6% and for Plateau State in 2012 was 4.8%.

Please download this article again to view the correct version. The originally published, uncorrected article and the republished, corrected article are provided here for reference.

## Supporting information

S1 File Originally published, uncorrected article. (PDF)

S2 File Republished, corrected article. (PDF)
