# Supplementary material for: An impact evaluation of two rounds of mass drug administration on the prevalence of active trachoma: A clustered cross sectional survey
Source: PLoS One. 2018 Aug 29;13(8):e0201911. doi: 10.1371/journal.pone.0201911 (PMC6114510; doi:10.1371/journal.pone.0201911)

**S1 File: Consent, Procedure and Confidentiality Information for Adult Study Participants**

This study received approval from Institutional Review Board of Emory University under

Paper Study Number 079-2006.

**Introduction:** You are being asked to volunteer for a research study to assess the impact of two rounds of MDA on trachoma prevalence in Plateau and Nasarawa States in Nigeria. We want to determine the impact of only two rounds of MDA on trachoma prevalence. You are being asked to participate in this project because you are involved in the delivery of mass drug administration or clinical examination for trachoma grading in Plateau or Nasarawa States. Your participation in this research is voluntary. You may choose not to participate at any time without penalty.

**Procedures:** Participation in this project consists of responding to questions about your involvement in trachoma and the drugs used for trachoma treatment. You may also be requested to have your children, if any, examined for trachoma. The questions will be posed by the PI and/or the study coordinator using a structured questionnaire. The interview should take approximately 15 minutes. The clinical examination of a child’s eyes will take about five minutes. You will be expected to respond honestly to questions asked, but all answers are considered correct and appropriate. Children younger than ten years old will have their eyes examined by a trained and qualified professional. You may come into contact with the following research team members: Carter Center district and country staff members, trained field investigators, and the principal investigator.

**Risks:** There are no foreseeable risks to you or your child from participation in this

project.

**Benefits:** Taking part in this research study may not benefit you personally, but we may learn new things that will help the Ministry of Health (MOH) in Nigeria, the Carter Center and the affected communities to achieve more efficient and effective trachoma interventions in order to eliminate and control these diseases in Nigeria. After the information has been collected and analyzed, the MOH will be provided with feedback on the results of the study.

**Confidentiality:** Your responses to interview questions will be kept confidential and will not be shared. People other than those doing the study may look at the study records. Agencies that make rules and policies about how research is done have the right to review these records. So do agencies that pay for the study. Those with the right to look at your study records include people at Emory University Rollins School of Public Health, the Emory University Institutional Review Board, and staff members of The Carter Center. Records can also be opened by court order. We may use a study number or your name on study records as may be appropriate. Your name will not be used in any data analysis. Your name and other facts that might point to you will not appear when we present this study or publish its results.

**Voluntary Participation and Withdrawal:** Your participation is voluntary and you have the right to refuse to be in this study. You and other subjects have the right to withdraw from the study at any time without penalty.

(Permission message)

I understand all the explanations and commitment above and therefore grant permission to the scientists and health workers from the MOH, Emory University and The Carter Center to work hand and hand with us in order to control trachoma in our community. We will give you a copy of this consent form to keep. I agree to participate in this study voluntarily.

Person giving consent

Signature ____________________________________________ Date

__________________

Person obtaining consent

Signature ____________________________________________ Date

__________________

**Contact Persons:** If you have any questions about this project, please contact the Principal Investigator, Dr Asrat Amnie, asrat.amnie@emory.edu or (+1 404 784 4796) or Dr Paul Emerson, pemerson@emory.edu .If you have any questions about your rights in this research, please contact Dr. James Keller, Chair of the Emory University Institutional Review Board, at (jim@radonc.emory.org ) or (+1 404 712 0720)


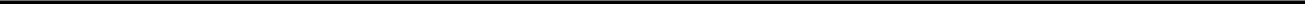

Supplement: S1 File — This is a statement of Informed Consent, outlining the procedure, benefits, risks, participant rights, confidentiality and voluntary participation in and withdrawal from the study. (DOC) [file pone.0201911.s001.doc]
