# Supplementary material for: An impact evaluation of two rounds of mass drug administration on the prevalence of active trachoma: A clustered cross sectional survey
Source: PLoS One. 2018 Aug 29;13(8):e0201911. doi: 10.1371/journal.pone.0201911 (PMC6114510; doi:10.1371/journal.pone.0201911)
Supplement: S2 File — Checklist of items that were included in reports of cross-sectional studies (DOC) [file pone.0201911.s002.doc]

S2 File. Checklist of Recommendations Done in the Study as per STROBE Statement

|  | Item No | Recommendations done |
| --- | --- | --- |
| **Title and abstract** | 1 | (*a*) The study’s design , Cross-sectional study, was indicated in the title |
|  |  | (*b*) The abstract is informative and balanced summary of what was done and what was found |
| Introduction | | |
| Background/rationale | 2 | The scientific background and rationale for the investigation being reported is done |
| Objectives | 3 | The specific objectives of the study are stated. |
| Methods | | |
| Study design | 4 | P key elements of study design are presented early in the paper |
| Setting | 5 | The setting, locations, and relevant dates, including periods of recruitment, and data collection were described in the method section of the manuscript. |
| Participants | 6 | (*a*) Given: the eligibility criteria, and the sources and methods of selection of participants |
| Variables | 7 | Confounding factors that would impact trachoma prevalence such as displacement of large number of people into or out of the study area ruled out |
| Data sources/ measurement | 8* | Data collection methods described and survey instruments provided as supporting information. |
| Bias | 9 | A week-long training provided for data collectors to address potential sources of bias |
| Study size | 10 | Adequate sample size obtained in proportion to the study population |
| Quantitative variables | 11 | A comparison of mean trachoma baseline and mean post intervention prevalence |
| Statistical methods | 12 | (*a*) The analysis was conducted using SAS version 9.3 |
|  |  | (*b*) Individual level responses from the census form were analyzed using SURVEYFREQ applying cluster-level weights. |
|  |  | (*c*) Cluster #30 was the only missing data area inaccessible because of security reasons. Missing data treated as missing |
|  |  | (*d* The CHISQ is the test of significance in the SURVEYFREQ procedure. |
|  |  | (*e*) A computational procedure , the Taylor series expansion method , was used to adjust for the design effects based on effective sample sizes to create adjusted estimates of confidence intervals using information on the strata of the sampling frame. |
| Results | | |
| Participants | 13* | (a) Individuals who consented to be interviewed for the study and have clinical eye exams are eligible as study participants |
|  |  | (b) missing the pre-survey announcements is reason for non-participation. |
|  |  | (c) use of a flow diagram not considered |
| Descriptive data | 14* | (a) Given: characteristics of study participants (eg demographic, clinical, social) and information on exposures and potential confounders |
|  |  | (b) Cluster # was the missing data. Approximately 15-20 household missed |
| Outcome data | 15* | Reported: numbers of outcome events or summary measures |
| Main results | 16 | (*a*) Given: adjusted estimates their precision (eg, 95% confidence interval). Design effect adjusted |
|  |  | (*b*) No risk analysis done in the study. |
|  |  | (*c*) I translating estimates of relative risk into absolute risk not aplicable |
| Other analyses | 17 | Report other analyses not applicable |
| Discussion | | |
| Key results | 18 | Summarized: key results with reference to study objectives |
| Limitations | 19 | Discussed: limitations of the study, taking into account sources of potential bias or imprecision. |
| Interpretation | 20 | Given: a cautious overall interpretation of results considering objectives, limitations, multiplicity of analyses, results from similar studies, and other relevant evidence |
| Generalizability | 21 | Discussed: the generalizability (external validity) of the study results |
| Other information | | |
| Funding | 22 | This is an ongoing trachoma prevention program. There is no funding provided to the investigator |
