# Supplementary material for: An impact evaluation of two rounds of mass drug administration on the prevalence of active trachoma: A clustered cross sectional survey
Source: PLoS One. 2018 Aug 29;13(8):e0201911. doi: 10.1371/journal.pone.0201911 (PMC6114510; doi:10.1371/journal.pone.0201911)
Supplement: S1 Table — The questionnaire was designed for mass drug administration coverage Survey and has the following sections: (1) Respondents Demographics (2) Household Possessions (HP); (3) Knowledge of Mass Drug Administration of participants (M); (4) Questions on Water Supply and Sanitation (WS); (5) Use of Mosquito Nets (MN); (6) Household Socioeconomic Status (ES); (7) Possession of Latrine (PL); (8) A chart to collect the results of eye examination by a trained eye care professional. The questions have been coded RD1, RD2, RD3, etc. to denote Respondents’ Demographics; HP3, HP4, HP5, to denote house Hold Possession. As this questionnaire is a subset of a larger questionnaire used in trachoma surveys, questions not relevant for this study were omitted (for example, HP1, and HP2). The questionnaire also consists of information not relevant for this manuscript such as the use of mosquito bed nets for malaria prevention. In the program areas, malaria prevention and trachoma prevention are jointly implemented. Information is saved into electronic tablets by field workers as they interview heads of the households and household members. (DOC) [file pone.0201911.s004.doc]

Supporting information

**S1 Table: Questionnaire for Nigeria MDA Coverage Survey, June 2012**

| Serial Number: | | | |  | Data Entry 1 | |__|__| |  | Date: | |
| --- | --- | --- | --- | --- | --- | --- | --- | --- | --- |
|  |  |  |  | |__|__|__|__|__| | *(initials)* |  |  |  |  |
|  |  |  |  |  |  |  |  |  |  |
|  |  |  |  |  |  |  |  |  |  |
| Cluster Number | | | |  | Data Entry 2 | |__|__| |  | Date: | |
|  |  |  |  |  |  |  |  |  |  |
| (1-40): | | |  | |__|__| | *(initials)* |  |  |  |  |
|  |  |  |  |  |  |  |  |  |  |
|  |  |  | |  |  |  | |  |  |
| Latitude | | | | |__|__| |__|__|__|__|__| | Longitude | |__|__|__| |__|__|__|__|__| | | | |
|  |  |  |  |  |  |  | |  |  |
|  | Village name | | |  | Survey Date (DD/MM/YYYY) | | | | |
|  |  |  |  |  | \|__\|__\|/\|__\|__\|/\|__\|__\|__\|__\| | | |  |  |
|  |  |  |  |  |  |  |  |  |  |
|  |  |  | |  |  | | | |  |
| Household | | | | |__|__| | Household interview consent given? No= 0 | | | | |
| number: | | | |  | Yes= 1 *(if no, END)* | |  |  |  |
|  |  |  |  |  |  |  |  |  |  |
|  |  |  | |  |  |  |  |  |  |
|  | Household Demographics | | | |  |  |  |  |  |
|  |  |  |  |  |  | | | |  |
| Co | |  |  |  | First malaria HH where more | | | |  |
|  |  |  | Describe this household in relation to the | | than one malaria HH per MDA | | | |  |
| mpo | |  |  |  | HH (includes head of MDA | | | |  |
|  |  |  | compound. |  |  |  |  |  |  |
| und | |  |  |  |  |  | HH)=1 | |  |
|  |  |  |  |  |  |  |  |  |  |
|  |  |  |  |  | Other malaria HH where there | | | |  |
| RD1 | |  | Name of respondent | |  |  |  |  |  |
|  |  |  |  |  |  | | | |  |
|  |  |  |  |  | Head of household=1 | | | |  |
| RD2 | |  | Description of Respondent | | Wife of head of household=2 | | | |  |
|  |  |  |  |  |  |  | Other=99 | |  |
|  | |  |  | |  | |  | |  |
| RD3 | |  | Gender of Respondent | | Male= 1 | | Female=0 | |  |
|  | |  |  | |  |  |  |  |  |
| RD4 | |  | How old are you? *(in years, round* | |  |  |  |  |  |
|  |  |  | *months down)* | |  |  |  |  |  |
|  |  |  |  |  |  |  |  |  |  |



|  | What is your ethnicity? *Write name of* | __________________________ |  |
| --- | --- | --- | --- |
| RD5 | *ethnicity* |  |  |
|  |  | ____ |  |
|  |  |  |  |
|  |  |  |  |
|  |  | Single & Never |  |
|  |  | Married………...1 |  |
|  |  | Married……………………… |  |
|  |  | ….2 |  |
|  | What is your current marital status? | Not Married Now-- |  |
| RD8 | *Do not read list* | Divorced or |  |
|  |  | Separated…………………..….. |  |
|  |  | .3 |  |
|  |  | Widowed……………………… |  |
|  |  | ..4 |  |
|  |  |  |  |
|  |  | None........................................... |  |
|  |  | .......0 |  |
|  |  | Primary |  |
|  |  | ...................………………..1 |  |
|  | What is the highest level of school | Junior |  |
| RD9 | anyone in your household has attained | secondary.............………...…2 |  |
|  | (started)? | Senior |  |
|  |  |  |  |
|  |  | secondary........……………...3 |  |
|  |  | College / |  |
|  |  | University……………......4 |  |
|  |  | Religious |  |
|  |  | school...............................5 |  |
|  |  |  |  |



|  |  |  |  | Farmer/cattle | | |  | |
| --- | --- | --- | --- | --- | --- | --- | --- | --- |
|  |  |  | rearing......................... | | 1 | |  | |
|  |  |  | Formal employment | | | |  | |
|  |  |  | (salary) | | .............2 | |  | |
|  |  |  |  |  | Trade | |  | |
|  |  |  | (business)................................. | | 3 | |  | |
|  |  | What is the main occupation of the head |  |  | Daily | |  | |
| RD1 | | of the household? | laborer.................................... | | 4 | |  | |
|  |  | **NOTE: this is the occupation of the** |  |  |  | |  | |
| 1 |  |  | Housewife.................................. | |  | |  | |
|  |  | **head of the malaria HH (wife in most** |  |  | ........5 | |  | |
|  |  | **cases)** | Other........................................... | |  | |  | |
|  |  |  |  |  |  |  |  |  |
|  |  |  |  |  | .....99 | |  | |
|  |  |  | *(specify)*:__________________ | | | |  | |
|  |  |  |  |  | ____ | |  | |
|  |  |  |  | | | |  | |
|  |  |  | No……………....…………… | | | |  | |
|  |  |  |  |  | ……..0 | |  | |
|  |  |  |  | Yes, but only | | |  | |
| RD1 | | Does this household own any land? | lease............................. | | 1 | |  | |
|  |  |  |  |  |  |  |  |  |
| 2 |  |  |  | Yes, and own | | |  | |
|  |  |  |  |  |  |  |  |  |
|  |  |  |  |  |  |  |  |  |
|  |  |  | outright........................ | | 2 | |  | |
|  |  |  |  |  | Do not | |  | |
|  |  |  | know……..…..……………88 | | | |  | |
|  |  |  |  |  |  | |  | |
| Household Possessions | | |  |  |  | |  | |
|  |  |  |  |  |  | |  | |
| HP3 |  | Does your household have a functioning |  | No=0; Yes=1 | | |  | |
|  |  | radio set? |  |  |  |  |  | |
|  |  |  |  |  |  |  |  |  |
|  |  |  |  | | | |  | |
| HP4 |  | Does your household have working | No=0; Yes=1; Yes, | | | |  | |
|  |  | electricity? |  | generator=2 | | |  | |
|  |  |  |  |  |  |  |  |  |
|  |  |  |  |  |  | |  | |
| HP5 |  | Does your household have a functioning |  | No=0; Yes=1 | | |  | |

MDA Knowledge and Participation


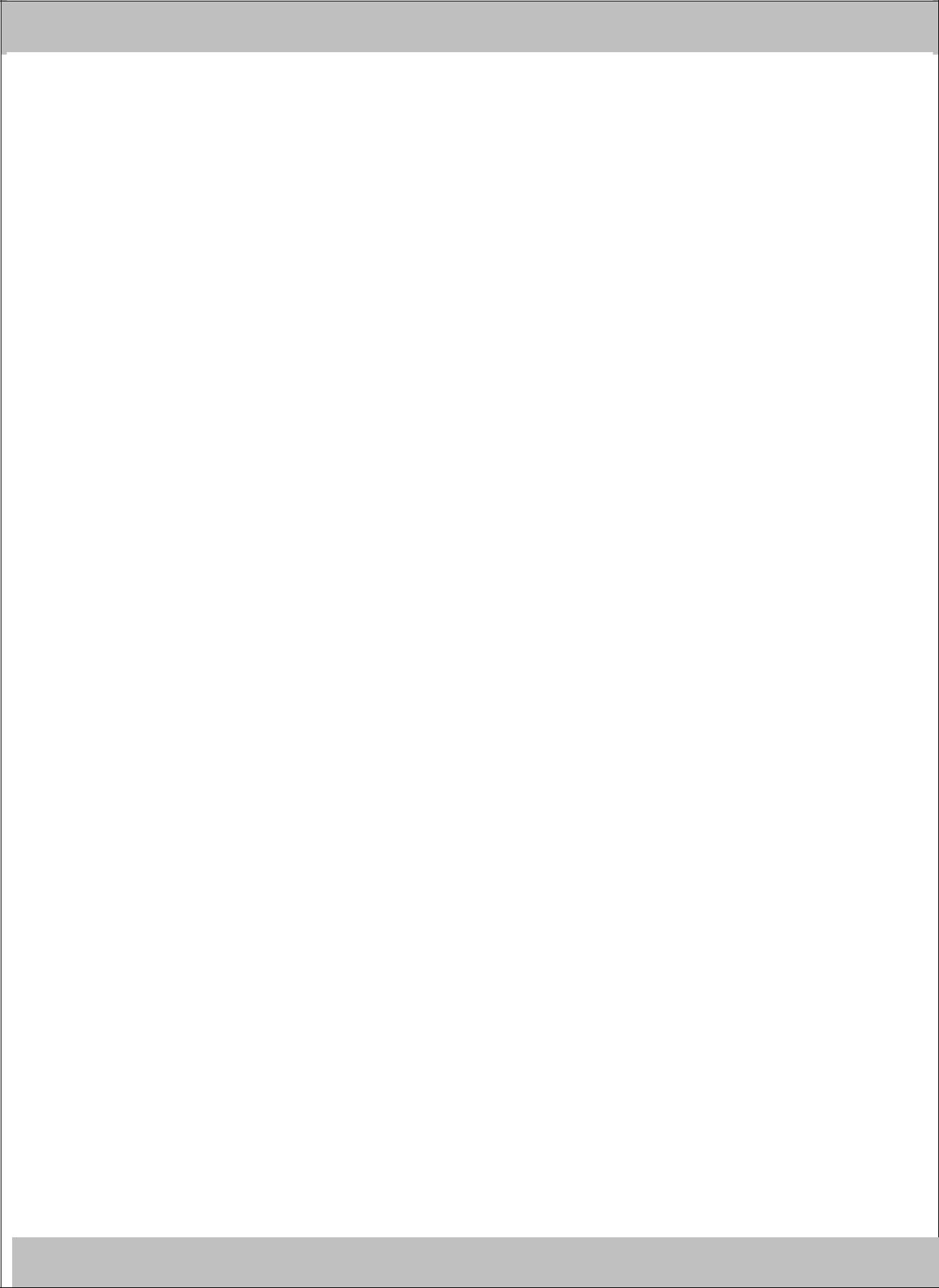


|  | What disease do these drugs treat? | Trachoma=1 |  |
| --- | --- | --- | --- |
|  |  |  |  |
|  | *(show Zithromax and Tetracycline)* | Lymphatic Filariasis=2 |  |
|  |  |  |  |
|  | **NOTE: Recorders showed measuring** | Onchocerciasis=3 |  |
|  | **stick, pink pills and empty pos bottle.** |  |  |
| M1 |  |  |  |
|  | **Clearly differentiated between pink** | Schistosomiasis=4 |  |
|  | **pills and other white pills distributed.** |  |  |
|  | **If person described trachoma but did** | I do not know=88 |  |
|  | **not know the name, it was counted as** | Other =99 |  |
|  | **trachoma. Just saying eye disease** |  |  |
|  | **should have been marked as other.** | (specify)__________________________ |  |
|  |  |  |  |
| M2 | Did your household get these drugs from | No=0; Yes=1 |  |
|  | a CDD? |  |  |
|  |  |  |  |
|  |  |  |  |
|  | Prior to the day of distribution, were you | No=0; Yes=1 |  |
| M3 | informed that these drugs would be given |  |  |
|  | out? |  |  |
|  |  |  |  |
| K4 | What can happen to a person who has |  |  |
|  | trachoma? |  |  |
|  | *(multiple response)* | Nothing happens=1 |  |
|  |  | Blindness=2 |  |
|  |  | Reduced vision=3 |  |
|  | (*Do not read list. After each response ask* | I do not know=88 |  |
|  |  | Other=99 (*specify*)__________________ |  |
|  | *‘anything else?’ and indicate all* |  |  |
|  | *responses given.)* |  |  |
|  |  |  |  |
| K5 | How can someone protect him/herself |  |  |
|  | from trachoma? | Face washing/hygiene=1 |  |
|  |  |  |  |
|  | *(multiple response)* | Take antibiotics or medicine=2 |  |
|  |  | Trichiasis surgery=3 |  |
|  |  | Keeping environment clean=4 |  |
|  |  | Using pit latrines=5 |  |
|  | (*Do not read list. After each response ask* | I do not know=88 |  |
|  |  | Other =99 |  |
|  | *‘*anything else?’ and *Indicate all* |  |  |
|  |  | (*specify*)_______________________ |  |
|  | *responses given)* |  |  |
|  |  |  |  |
|  |  |  |  |
| K6 | Do you believe that trachoma is a | No=0; Yes=1; I don’t know=88 |  |
|  | problem in your community? |  |  |
|  |  |  |  |


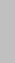
Water and Sanitation

|  |  |  | Unprotected spring=1 |  |
| --- | --- | --- | --- | --- |
|  |  |  | Protected spring=2 |  |
|  |  |  | Unprotected dug well=3 |  |
|  |  |  | Hand pump/tube well/borehole=4 |  |
|  |  | What is the main source of water your | Surface water (river, dam, lake, stream, |  |
|  | WS1 |  | canal)=5 |  |
|  |  | household uses for drinking? |  |  |
|  |  |  | Public piped water/tap/standpipe=6 |  |
|  |  |  |  |  |
|  |  |  | Private piped into yard/dwelling=7 |  |
|  |  |  | Rainwater collection=8 |  |
|  |  |  | Other=99 |  |
|  |  |  | *(Specify)_________________* |  |
|  |  |  |  |  |
|  |  | How long does a roundtrip take for you | Less than 30 min=1 |  |
|  |  |  |  |  |
|  | WS2 | to collect water used for drinking – | 30 min to 1 hour=2 |  |
|  |  | including time to walk there, collect |  |  |
|  |  |  |  |  |
|  |  | water and return? | More than one hour=3 |  |
|  |  |  |  |  |
|  |  |  |  |  |
|  |  |  |  | If |
|  | WS3 | Is the water source you use for drinking | No=0; Yes=1 | WS3=1, |
|  |  | the same you use for bathing? |  | go to |
|  |  |  |  |  |
|  |  |  |  | ES7 |
|  |  |  |  |  |
|  |  | If no, how long does a roundtrip take for | Less than 30 min=1 |  |
|  |  |  |  |  |
|  | WS4 | you to collect water used for bathing – | 30 min to 1 hour=2 |  |
|  |  | including time to walk there, collect |  |  |
|  |  |  |  |  |
|  |  | water and return? | More than one hour=3 |  |
|  |  |  |  |  |
|  |  |  |  |  |
|  | Mosquito nets | |  |  |
|  |  |  |  |  |
|  | MN1 | How many dependents do you have who | ___________________ |  |
|  |  | are living with you here? |  |  |
|  |  |  |  |  |
|  |  |  |  |  |
|  | MN2 | How many sleeping spaces do you have | ___________________ |  |
|  |  | for you and your dependents? |  |  |
|  |  |  |  |  |
|  |  |  |  |  |
|  |  |  |  |  |
|  |  |  |  | If |
|  | MN3 | Do you have any mosquito nets in this | ___________________ | MN3=0 |
|  |  | household? |  | then skip |
|  |  |  |  |  |
|  |  |  |  | to ES7 |
|  |  |  |  |  |
|  | MN4 | How many total mosquito nets do you | ___________________ |  |
|  |  |  |  |  |
|  |  |  |  |  |

|  |  |  | have for you and your dependents? |  |  |  |  |
| --- | --- | --- | --- | --- | --- | --- | --- |
|  |  |  |  |  |  |  |  |
|  |  |  | How many of these nets were given to |  |  |  |  |
|  | MN5 | | you during a campaign Dec 2010/Jan |  | ___________________ | |  |
|  |  |  | 2011? |  |  |  |  |
|  |  |  |  |  |  | |  |
|  | MN6 | | How many nets are hanging now? |  | ___________________ | |  |
|  |  |  |  |  |  | |  |
|  | MN7 | | How many nets were used last night? |  | ___________________ | |  |
|  |  |  |  |  |  |  |  |
|  | MN8 | | How many of the reported nets did you |  | ___________________ | |  |
|  |  |  | observe? |  |  |  |  |
|  |  |  |  |  |  |  |  |
|  |  |  |  |  |  |  |  |
|  | Observations | | | |  |  |  |
|  |  |  |  |  |  | | |
|  |  |  | What is the main construction material |  | Thatch=1 | |  |
|  |  |  |  |  |  |  |  |
|  |  |  | for the roof? |  | Stick and mud=2 | |  |
|  | ES7 |  | **NOTE: If there were multiple** |  | Corrugation iron/metal=3 | |  |
|  |  |  | **materials, the “highest ses” selected.** |  |  |  |  |
|  |  |  | **E.g. if most thatch and one iron, iron** |  | Other=99 | |  |
|  |  |  | **selected.** |  | *(specify)_______________* | |  |
|  |  |  |  |  |  |  |  |
|  |  |  |  |  |  |  |  |
|  | ES8 |  | Are cattle kept within 10 meters of the |  | No=0; Yes=1; Do not keep cattle=2 | |  |
|  |  |  | living spaces in this HH? |  |  |  |  |
|  |  |  |  |  |  |  |  |
|  |  |  |  |  |  |  |  |
|  |  |  | Is there a household latrine (*observed*)? |  |  |  | If |
|  |  |  |  |  |  |  | PL1=0, |
|  |  |  |  |  |  |  |  |
|  | PL1 |  | **NOTE: If latrine was collapsed,** |  | No=0; Yes=1 | | skip next |
|  |  |  | **marked “no”** |  |  |  | two |
|  |  |  |  |  |  |  | questions |
|  |  |  |  |  |  |  |  |
|  |  |  |  |  |  |  |  |
|  | PL2 |  | Evidence of latrine usage observed |  | No=0; Yes=1 | |  |
|  |  |  | (*faeces in pit)?* |  |  |  |  |
|  |  |  |  |  |  |  |  |
|  |  |  |  |  | |  |  |
|  |  |  |  | Pit latrine without slab or open pit............ | | 1 |  |
|  |  |  |  | Pit latrine with slab.................................... | | 2 |  |
|  | PL3 |  | What type of latrine is present | Ventilated improved pit latrine (VIP)......... | | 3 |  |
|  |  |  |  | Flush or pour flush toilet............................ | | 4 |  |
|  |  |  |  |  |  |  |  |
|  |  |  |  | Public or shared sanitation facilities........... | | 5 |  |
|  |  |  |  | Other | .......................................................... | 99 |  |

| I | FIRS | S | AG | P | SLE | ANTIBIOTICS FOR | | | | |  |  |  |  |  |  |  |  |  |  |  |  |  |
| --- | --- | --- | --- | --- | --- | --- | --- | --- | --- | --- | --- | --- | --- | --- | --- | --- | --- | --- | --- | --- | --- | --- | --- |
| D | T | E | E In | RE | PT | TRACHOMA: HAVE | | | | | |  |  |  |  |  |  |  |  |  |  |  |  |
|  | NAM | X | yea | SE | UN | YOU EVER TAKEN | | | | |  |  |  |  |  |  |  |  |  |  |  |  |  |
|  |  |  |  |  |  | THESE DRUGS TAKEN | | | | | |  |  |  |  |  |  |  |  |  |  |  |  |
| # | E |  | rs/ | N | DER |  |  |  |  |  |  |  |  |  |  |  |  |  |  |  |  |  |  |
|  |  |  |  |  |  | TO YOU BY THE | | | | |  |  |  |  |  |  |  |  |  |  |  |  |  |
|  |  |  | wri | T |  |  |  |  |  |  |  |  |  |  |  |  |  |  |  |  |  |  |  |
|  |  |  |  |  | BED | CDD?NO=0 YES=1 | | | | |  | CLEAN | |  | RIGHT EYE | | |  |  | LEFT | | EYE |  |
|  |  |  | te 0 |  |  |  |  |  |  |  |  |  |  |  |  |  |  |  |  |  |  |  |  |
|  |  |  |  |  |  |  |  |  |  |  |  | FACE | |  |  |  |  |  |  |  |  |  |  |
|  |  |  | if |  | NET | **ZITHROMAX** | | | | |  |  |  |  |  |  |  |  |  |  |  |  |  |
|  |  |  |  |  |  |  |  |  |  |  |  |  |  |  |  |  |  |  |  |  |  |  |  |
|  |  |  | <1y |  | LAS | **TAB/SYRUP(ZMX)** | | | | |  |  |  |  |  |  |  |  |  |  |  |  |  |
|  |  |  | r/ |  | T | **TTC EYE** | | |  |  |  |  |  |  |  |  |  |  |  |  |  |  |  |
|  |  |  |  |  | NIG |  |  |  |  |  |  |  |  |  |  |  |  |  |  |  |  |  |  |
|  |  |  |  |  |  |  |  |  |  |  |  |  |  |  |  |  |  |  |  |  |  |  |  |
|  |  |  |  |  |  | **OINTMENT(TEO)** | | | | |  |  |  |  |  |  |  |  |  |  |  |  |  |
|  |  |  |  |  | HT? |  |  |  |  |  |  |  |  |  |  |  |  |  |  |  |  |  |  |
|  |  |  |  |  |  |  |  |  |  |  |  |  |  |  |  |  |  |  |  |  |  |  |  |
|  |  |  |  |  |  |  |  |  |  |  |  |  |  |  |  |  |  |  |  |  |  |  |  |
|  |  |  |  |  |  | Z | T |  | WHY |  | VE | OC | NA | T | T | T | T | C | T | T | T | T | C |
|  |  |  |  |  |  | M | E |  | NOT |  | RI- | UL | SA | F | I | S | T | O | F | I | S | T | O |
|  |  |  |  |  |  | X | O |  | REFUS |  | FIE | AR | L |  |  |  |  |  |  |  |  |  |  |
|  |  |  |  |  |  |  |  |  | ED=1 |  | D | No | No |  |  |  |  |  |  |  |  |  |  |
|  |  |  |  |  |  |  |  |  |  |  | NO |  |  |  |  |  |  |  |  |  |  |  |  |
|  |  |  |  |  |  |  |  |  | MISSE |  |  | =0 | =0 |  |  |  |  |  |  |  |  |  |  |
|  |  |  |  |  |  |  |  |  |  |  | =0 |  |  |  |  |  |  |  |  |  |  |  |  |
|  |  |  |  |  |  |  |  |  | D=2 |  |  |  | YE |  |  |  |  |  |  |  |  |  |  |
|  |  |  |  |  |  |  |  |  |  |  | YES | YE |  |  |  |  |  |  |  |  |  |  |  |
|  |  |  |  |  |  |  |  |  |  |  |  |  | S= |  |  |  |  |  |  |  |  |  |  |
|  |  |  |  |  |  |  |  |  |  |  | =1 |  |  |  |  |  |  |  |  |  |  |  |  |
|  |  |  |  |  |  |  |  |  | NOT |  |  | S= |  |  |  |  |  |  |  |  |  |  |  |
|  |  |  |  |  |  |  |  |  |  |  |  |  | 1 |  |  |  |  |  |  |  |  |  |  |
|  |  |  |  |  |  |  |  |  |  |  | NO |  |  |  |  |  |  |  |  |  |  |  |  |
|  |  |  |  |  |  |  |  |  | DISTRI |  |  | 1 |  |  |  |  |  |  |  |  |  |  |  |
|  |  |  |  |  |  |  |  |  |  |  |  |  |  |  |  |  |  |  |  |  |  |  |  |
|  |  |  |  |  |  |  |  |  |  |  | REC |  |  |  |  |  |  |  |  |  |  |  |  |
|  |  |  |  |  |  |  |  |  | BUTE |  |  |  |  |  |  |  |  |  |  |  |  |  |  |
|  |  |  |  |  |  |  |  |  |  |  | OR |  |  |  |  |  |  |  |  |  |  |  |  |
|  |  |  |  |  |  |  |  |  | D=3 |  |  |  |  |  |  |  |  |  |  |  |  |  |  |
|  |  |  |  |  |  |  |  |  |  |  | D= |  |  |  |  |  |  |  |  |  |  |  |  |
|  |  |  |  |  |  |  |  |  |  |  |  |  |  |  |  |  |  |  |  |  |  |  |  |
|  |  |  |  |  |  |  |  |  |  |  | 3 |  |  |  |  |  |  |  |  |  |  |  |  |
|  |  |  |  |  |  |  |  |  |  |  |  |  |  |  |  |  |  |  |  |  |  |  |  |
| 1 |  |  |  |  |  |  |  |  |  |  |  |  |  |  |  |  |  |  |  |  |  |  |  |
|  |  |  |  |  |  |  |  |  |  |  |  |  |  |  |  |  |  |  |  |  |  |  |  |
| 2 |  |  |  |  |  |  |  |  |  |  |  |  |  |  |  |  |  |  |  |  |  |  |  |
|  |  |  |  |  |  |  |  |  |  |  |  |  |  |  |  |  |  |  |  |  |  |  |  |
| 3 |  |  |  |  |  |  |  |  |  |  |  |  |  |  |  |  |  |  |  |  |  |  |  |
|  |  |  |  |  |  |  |  |  |  |  |  |  |  |  |  |  |  |  |  |  |  |  |  |
| 4 |  |  |  |  |  |  |  |  |  |  |  |  |  |  |  |  |  |  |  |  |  |  |  |
|  |  |  |  |  |  |  |  |  |  |  |  |  |  |  |  |  |  |  |  |  |  |  |  |
| 5 |  |  |  |  |  |  |  |  |  |  |  |  |  |  |  |  |  |  |  |  |  |  |  |
|  |  |  |  |  |  |  |  |  |  |  |  |  |  |  |  |  |  |  |  |  |  |  |  |
| 6 |  |  |  |  |  |  |  |  |  |  |  |  |  |  |  |  |  |  |  |  |  |  |  |
|  |  |  |  |  |  |  |  |  |  |  |  |  |  |  |  |  |  |  |  |  |  |  |  |
| 7 |  |  |  |  |  |  |  |  |  |  |  |  |  |  |  |  |  |  |  |  |  |  |  |
|  |  |  |  |  |  |  |  |  |  |  |  |  |  |  |  |  |  |  |  |  |  |  |  |
| 8 |  |  |  |  |  |  |  |  |  |  |  |  |  |  |  |  |  |  |  |  |  |  |  |
|  |  |  |  |  |  |  |  |  |  |  |  |  |  |  |  |  |  |  |  |  |  |  |  |
| 9 |  |  |  |  |  |  |  |  |  |  |  |  |  |  |  |  |  |  |  |  |  |  |  |
|  |  |  |  |  |  |  |  |  |  |  |  |  |  |  |  |  |  |  |  |  |  |  |  |
| 1 |  |  |  |  |  |  |  |  |  |  |  |  |  |  |  |  |  |  |  |  |  |  |  |
| 0 |  |  |  |  |  |  |  |  |  |  |  |  |  |  |  |  |  |  |  |  |  |  |  |
