# Supplementary material for: An impact evaluation of two rounds of mass drug administration on the prevalence of active trachoma: A clustered cross sectional survey
Source: PLoS One. 2018 Aug 29;13(8):e0201911. doi: 10.1371/journal.pone.0201911 (PMC6114510; doi:10.1371/journal.pone.0201911)
Supplement: S2 Table — This consists of information on the Local Government Areas (LGA) of the two states of Plateau and Nasarawa. Each LGA is divided into several Enumeration Areas (used for census purposes in Nigeria) which are assigned cluster numbers. (DOC) [file pone.0201911.s005.doc]

**S2 Table: List of LGAs and Enumeration Areas of the Study Area and Assigned Cluster Numbers**

| **Seria** | **State** | **LGA** |  | **Locality** |  |  | **EA No.** | **cluster** |
| --- | --- | --- | --- | --- | --- | --- | --- | --- |
| **l** |  |  |  | **code** | **Locality Name** |  |  | **no** |
| **64** | Plateau | Shendam |  | 000075001 | TENGZET(GUNG) |  | 0132 | 1 |
| **207** | Plateau | Shendam | 000056018 | | ANG.NBUAB(SHENDAM | | 0480 | 2 |
| **350** | Plateau | Shendam |  | 000038000 | RINGLONG | | 0652 | 3 |
| **492** | Plateau | Shendam | 000029000 | | YELWA | | 0954 | 4 |
| **635** | Plateau | Shendam |  | 000074001 | NGRAS (DANGAT) | | 1240 | 5 |
| **778** | Plateau | Shendam | 000032007 | | DUNGKAKSAK | | 1524 | 6 |
| **921** | Plateau | Shendam |  | 000004010 | KADONG | | 1810 | 7 |
| **1064** | Plateau | Shendam | 000084001 | | ANGWAN MUSA | | 2092 | 8 |
| **1206** | plateau | Langtang |  | 000065000 | LANGAN | | 0116 | 9 |
|  |  | N |  |  |  |  |  |  |
| **1349** | plateau | Langtang | 000029004 | | GUNUNG (ZABAI LAKA) | | 0414 | 10 |
|  |  | N |  |  |  |  |  |  |
| **1492** | plateau | Langtang |  | 000038002 | WARRANG | | 0698 | 11 |
|  |  | N |  |  |  |  |  |  |
| **1635** | plateau | Langtang | 000020000 | | PISHE | | 0986 | 12 |
|  |  | N |  |  |  |  |  |  |
| **1778** | plateau | Langtang |  | 000011000 | LANGTANG | | 1272 | 13 |
|  |  | N |  |  |  |  |  |  |
| **1920** | plateau | Langtang | 000010000 | | BALLZE | | 1556 | 14 |
|  |  | N |  |  |  |  |  |  |
| **2063** | plateau | Langtang |  | 000013000 | INYERGBRMAN | | 1862 | 15 |
|  |  | N |  |  |  |  |  |  |
| **2206** | Plateau | wase | 000063014 | | ANG TUDUN SAFIYO | | 0248 | 16 |
| **2349** | Plateau | wase |  | 000016004 | DAURA | | 0528 | 17 |
| **2492** | Plateau | wase | 000027015 | | MALLAN ADAMA | | 0808 | 18 |
| **2634** | Plateau | wase |  | 000017002 | NAKIRAINA(BANGALA | | 1084 | 19 |
|  |  |  |  |  | LA) | |  |  |
| **2777** | Plateau | wase | 000028000 | | DEMSUN | | 1362 | 20 |
| **42** | Nasaraw | Doma |  | 000014000 | ANGWAN YARA | | 0092 | 1 |
|  | a |  |  |  |  |  |  |  |
| **158** | Nasaraw | Doma | 000043000 | | ACHALAGU | | 0888 | 2 |
|  | a |  |  |  |  |  |  |  |
| **275** | Nasaraw | Doma |  | 000066006 | AKPOKWU GBEJI | | 1248 | 3 |
|  | a |  |  |  |  |  |  |  |
| **391** | Nasaraw | Doma | 000073016 | | ZEVER | | 1442 | 4 |
|  | a |  |  |  |  |  |  |  |
| **507** | Nasaraw | Doma |  | 000092000 | IGBABO | | 1750 | 5 |
|  | a |  |  |  |  |  |  |  |
| **623** | Nasaraw | Obi | 000013003 | | RUGAN USMAN | | 0100(PARTIA | 6 |
|  | a |  |  |  | KICHEME | | L) |  |


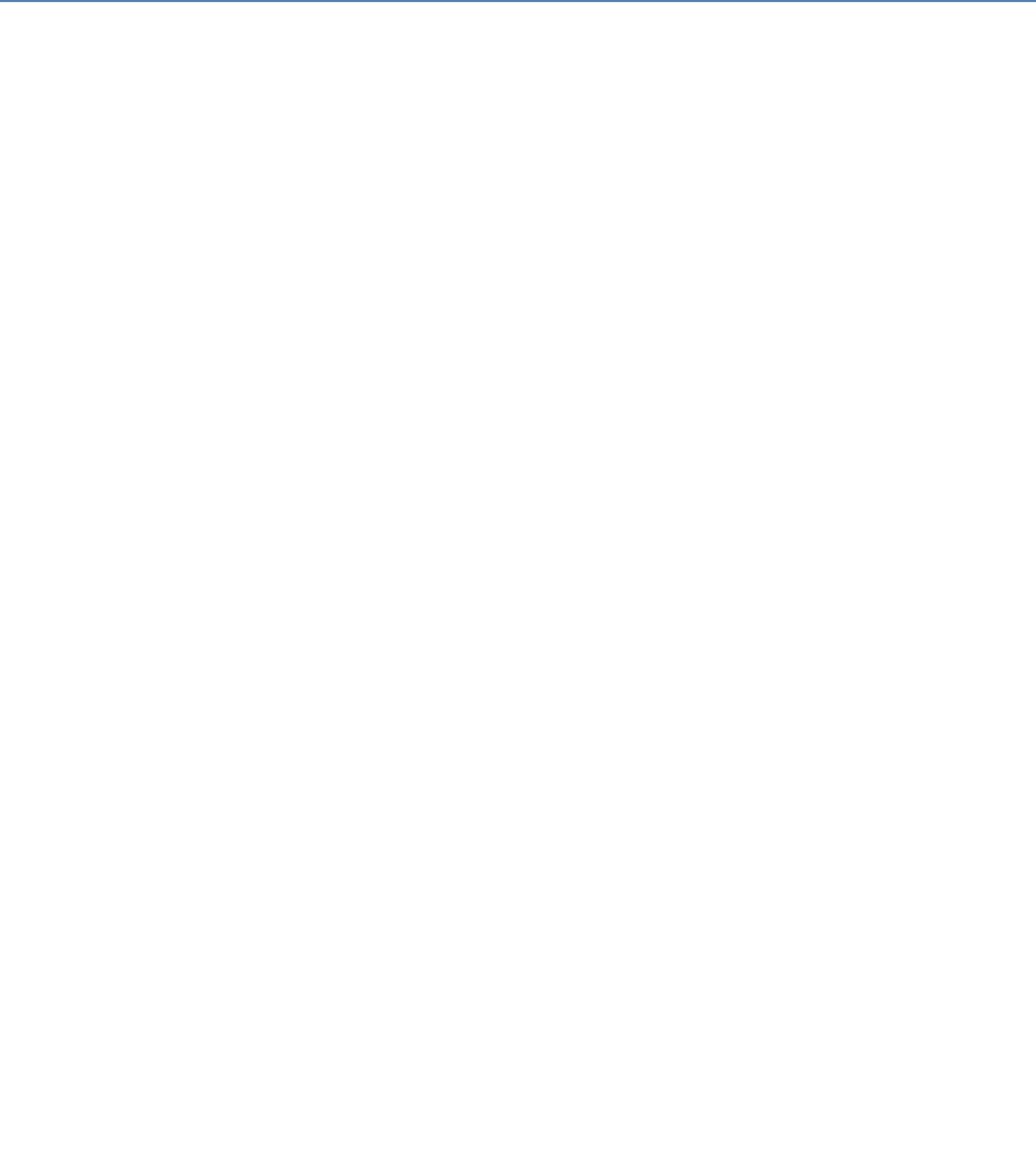


| **740** | Nasaraw | Obi |  | 000022000 | AGYARAGU | 0332 | 7 |
| --- | --- | --- | --- | --- | --- | --- | --- |
|  | a |  |  |  |  |  |  |
| **856** | Nasaraw | Obi | 000045000 | | OME | 0564 | 8 |
|  | a |  |  |  |  |  |  |
| **972** | Nasaraw | Obi |  | 000059000 | DADDARE | 0834 | 9 |
|  | a |  |  |  |  |  |  |
| **1088** | Nasaraw | Obi | 000066000 | | OBI | 1026 | 10 |
|  | a |  |  |  |  |  |  |
| **1205** | Nasaraw | Obi |  | 000095003 | PETER AKAHA | 1266 | 11 |
|  | a |  |  |  |  |  |  |
| **1321** | Nasaraw | Obi | 000130000 | | GBERWUA | 1498 | 12 |
|  | a |  |  |  |  |  |  |
| **1437** | Nasaraw | Keana |  | 000024000 | ANGWAN DOGO | 0196 | 13 |
|  | a |  |  |  |  |  |  |
| **1553** | Nasaraw | Keana | 000096000 | | AGBARAGBA | 0544 | 14 |
|  | a |  |  |  |  |  |  |
| **1670** | Nasaraw | Awe |  | 000003000 | JANGWA | 0008 | 15 |
|  | a |  |  |  |  |  |  |
| **1786** | Nasaraw | Awe | 000006000 | | AZARA | 0240 | 16 |
|  | a |  |  |  |  |  |  |
| **1902** | Nasaraw | Awe |  | 000062000 | ADUNIYA | 0470 | 17 |
|  | a |  |  |  |  |  |  |
| **2018** | Nasaraw | Awe | 000084000 | | TUNGA | 0654 | 18 |
|  | a |  |  |  |  |  |  |
| **2135** | Nasaraw | Awe |  | 000118000 | KEKURA | 0798 | 19 |
|  | a |  |  |  |  |  |  |
|  |  |  |  |  |  |  |
| **2251** | Nasaraw | Awe | 000132001 | | USSER | 0996 | 20 |
|  | a |  |  |  |  |  |  |
