# Supplementary material for: An impact evaluation of two rounds of mass drug administration on the prevalence of active trachoma: A clustered cross sectional survey
Source: PLoS One. 2018 Aug 29;13(8):e0201911. doi: 10.1371/journal.pone.0201911 (PMC6114510; doi:10.1371/journal.pone.0201911)
Supplement: S3 Table — A total of 40 enumeration areas were selected by systematic random sampling. Each enumeration area is assigned a cluster number, and is divided into a weighted number of segments based on population density. Each enumeration area. is noted if it has a CDD (a Community Directed Drug Distributor assigned). Each segment may have as many as 15–20 household selected for study. (DOC) [file pone.0201911.s006.doc]

**S3 Table: Cluster Selected for Study by Systematic Random Sampling in Each Enumeration Area**

|  |  |  | **Localit** |  |  |  |  | **On** | **No.** |
| --- | --- | --- | --- | --- | --- | --- | --- | --- | --- |
| **Ser** |  |  |  |  |  | **Cluste** | **CDD** | **MDA** | **Segment** |
|  | **State** | **LGA** | **y code** | **Locality Name** | **EA No.** |  |  |  |  |
| **ial** |  |  |  |  |  | **r no** | **present** | **List** | **s** |
|  |  |  | 00007 | TENGZET(GUNG | 0132 |  |  | same |  |
|  |  |  | 5001 | ) |  |  |  | as |  |
|  |  |  |  |  |  |  |  | Gungl |  |
|  |  |  |  |  |  |  |  | ong or |  |
|  | Platea | Shend |  |  |  | **1** |  | dungu |  |
| 64 | u | am |  |  |  |  | No | ng? | 2 |
|  |  |  | 00005 | ANG.NBUAB(SH | 0480 |  |  | same |  |
|  |  |  | 6018 | ENDAM |  |  |  | as |  |
|  |  |  |  |  |  |  |  | PHC |  |
|  | Platea | Shend |  |  |  | **2** |  | Shen |  |
| 207 | u | am |  |  |  |  | yes | dam | 3 |
|  | Platea | Shend | 00003 | RINGLONG | 0652 | **3** |  |  |  |
| 350 | u | am | 8000 |  |  |  | yes | no | 2 |
|  |  |  | 00002 | YELWA | 0954 |  |  | same |  |
|  |  |  | 9000 |  |  |  |  | as |  |
|  |  |  |  |  |  |  |  | Marke |  |
|  |  |  |  |  |  |  |  | t |  |
|  | Platea | Shend |  |  |  | **4** |  | Yelwa |  |
| 492 | u | am |  |  |  |  | Yes | ? | 2 |
|  | Platea | Shend | 00007 | NGRAS | 1240 | **5** |  |  |  |
| 635 | u | am | 4001 | (DANGAT) |  |  | no | no | 1 |
|  | Platea | Shend | 00003 | DUNGKAKSAK | 1524 | **6** |  |  |  |
| 778 | u | am | 2007 |  |  |  | no | no | 1 |
|  | Platea | Shend | 00000 | KADONG | 1810 | **7** |  |  |  |
| 921 | u | am | 4010 |  |  |  | no | no | 1 |
|  |  |  | 00008 | ANGWAN MUSA | 2092 |  |  | same |  |
|  |  |  | 4001 |  |  |  |  | as |  |
|  |  |  |  |  |  |  |  | Ung. |  |
| 106 | Platea | Shend |  |  |  | **8** |  | Musa |  |
| 4 | u | am |  |  |  |  | yes | ? | 1 |
| 120 | platea | Langta | 00006 | LANGAN | 0116 | **9** |  |  |  |
| 6 | u | ngN | 5000 |  |  |  | yes | no | 1 |
| 134 | platea | Langta | 00002 | GUNUNG (ZABAI | 0414 | **10** |  |  |  |
| 9 | u | ngN | 9004 | LAKA) |  |  | yes | yes | 1 |
| 149 | platea | Langta | 00003 | WARRANG | 0698 | **11** |  |  |  |
| 2 | u | ngN | 8002 |  |  |  | no | no | 1 |
| 163 | platea | Langta | 00002 | PISHE | 0986 | **12** |  |  |  |
| 5 | u | ngN | 0000 |  |  |  | yes | yes | 1 |
| 177 | platea | Langta | 00001 | LANGTANG | 1272 | **13** |  |  |  |
| 8 | u | ngN | 1000 |  |  |  | yes | no | 4 |


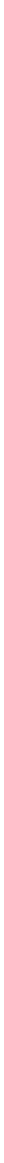


| 192 | platea | Langta | 00001 | BALLZE | 1556 | **14** |  |  |  |
| --- | --- | --- | --- | --- | --- | --- | --- | --- | --- |
| 0 | u | ngN | 0000 |  |  |  | yes | yes | 1 |
| 206 | platea | Langta | 00001 | INYERGBRMAN | 1862 | **15** |  |  |  |
| 3 | u | ngN | 3000 |  |  |  | yes | yes | 3 |
| 220 | Platea |  | 00006 | ANG TUDUN | 0248 | **16** |  |  |  |
| 6 | u | wase | 3014 | SAFIYO |  |  | no | yes | 1 |
| 234 | Platea |  | 00001 | DAURA | 0528 | **17** |  |  |  |
| 9 | u | wase | 6004 |  |  |  | no | yes | 1 |
| 249 | Platea |  | 00002 | MALLAN ADAMA | 0808 | **18** |  |  |  |
| 2 | u | wase | 7015 |  |  |  | yes | yes | 5 |
| 263 | Platea |  | 00001 | NAKIRAINA(BAN | 1084 | **19** |  |  |  |
| 4 | u | wase | 7002 | GALALA) |  |  | yes | yes | 3 |
| 277 | Platea |  | 00002 | DEMSUN | 1362 | **20** |  |  |  |
| 7 | u | wase | 8000 |  |  |  | yes | no | 3 |
|  | Nasar |  | 00001 |  |  | **21** |  |  |  |
| 42 | awa | Doma | 4000 | ANGWAN YARA | 0092 |  | no | no | 1 |
|  | Nasar |  | 00004 |  |  | **22** |  |  |  |
| 158 | awa | Doma | 3000 | ACHALAGU | 0888 |  | No | no | 1 |
|  | Nasar |  | 00006 | AKPOKWU |  | **23** |  |  |  |
| 275 | awa | Doma | 6006 | GBEJI | 1248 |  | No | no |  |
|  | Nasar |  | 00007 |  |  | **24** |  |  |  |
| 391 | awa | Doma | 3016 | ZEVER | 1442 |  | No | yes |  |
|  | Nasar |  | 00009 |  |  | **25** |  |  |  |
| 507 | awa | Doma | 2000 | IGBABO | 1750 |  | No | yes | 2 |
|  | Nasar |  | 00001 | RUGAN USMAN | 0100(PA | **26** |  |  |  |
| 623 | awa | Obi | 3003 | KICHEME | RTIAL) |  | No | no | 2 |
|  | Nasar |  | 00002 |  |  | **27** |  |  |  |
| 740 | awa | Obi | 2000 | AGYARAGU | 0332 |  | yes | no | 6 |
|  | Nasar |  | 00004 |  |  | **28** |  |  |  |
| 856 | awa | Obi | 5000 | OME | 0564 |  | yes | yes | 3 |
|  | Nasar |  | 00005 |  |  | **29** |  |  |  |
| 972 | awa | Obi | 9000 | DADDARE | 0834 |  | yes | yes | 1 |
| 108 | Nasar |  | 00006 |  |  | **30** |  |  |  |
| 8 | awa | Obi | 6000 | OBI | 1026 |  | yes | yes | 1 |
| 120 | Nasar |  | 00009 |  |  | **31** |  |  |  |
| 5 | awa | Obi | 5003 | PETER AKAHA | 1266 |  | No | no | 3 |
| 132 | Nasar |  | 00013 |  |  | **32** |  |  |  |
| 1 | awa | Obi | 0000 | GBERWUA | 1498 |  | yes | no | 2 |
| 143 | Nasar |  | 00002 |  |  | **33** |  |  |  |
| 7 | awa | Keana | 4000 | ANGWAN DOGO | 0196 |  | yes | no | 1 |
| 155 | Nasar |  | 00009 |  |  | **34** |  |  |  |
| 3 | awa | Keana | 6000 | AGBARAGBA | 0544 |  | yes | yes | 3 |
| 167 | Nasar |  | 00000 |  |  | **35** |  |  |  |
| 0 | awa | Awe | 3000 | JANGWA | 0008 |  | yes | yes | 2 |
| 178 | Nasar |  | 00000 |  |  | **36** |  |  |  |
| 6 | awa | Awe | 6000 | AZARA | 0240 |  | yes | yes | 1 |
| 190 | Nasar |  | 00006 |  |  | **37** |  |  |  |
| 2 | awa | Awe | 2000 | ADUNIYA | 0470 |  | No | no |  |
| 201 | Nasar |  | 00008 |  |  | **38** |  |  |  |
| 8 | awa | Awe | 4000 | TUNGA | 0654 |  | yes | yes |  |
| 213 | Nasar |  | 00011 |  |  | **39** |  |  |  |
| 5 | awa | Awe | 8000 | KEKURA | 0798 |  | yes | yes | 1 |
| 225 | Nasar |  | 00013 |  |  | **40** |  |  |  |
| 1 | awa | Awe | 2001 | USSER | 0996 |  | No | no | 1 |
