# Supplementary material for: An impact evaluation of two rounds of mass drug administration on the prevalence of active trachoma: A clustered cross sectional survey
Source: PLoS One. 2018 Aug 29;13(8):e0201911. doi: 10.1371/journal.pone.0201911 (PMC6114510; doi:10.1371/journal.pone.0201911)
Supplement: S4 Table — This is the schedule of a 5-day training of trachoma graders, and the use of electronic tablets for data collection. (DOC) [file pone.0201911.s007.doc]

**S4 Table: Training Agenda: Trachoma Impact Evaluation Plateau and Nasarawa States Tuesday, 5 June, 2012**

| 8:30 | Registration | Facilitators |
| --- | --- | --- |
| 9:00 | Welcome and Introductions | **Dr. Nimzing** |
| 9:15 | Opening Remarks | **Dr. E S Miri** |
| 9:30 | Purpose of training | **Lisa Dickman** |

9:45 Trachoma overview and SAFE strategy

**Dr. Nimzing**

10:15 Purpose of Surveys – describe protocol, define roles and responsibilities

**Lisa /Dr. Nimzing**

10:30 Tea break

10:45 Sampling, Village Protocol, Interviewing Best Practices

Distribute selected cluster list and discuss guidelines for household sampling to Recorders

| **Time** | **1. Trachoma Graders –Pateh** | **2. Recorders – NAME Lisa/Nimzing** |
| --- | --- | --- |
|  | **Makalo/Nimzing** |  |
|  |  |  |
| 11:00 – | Anatomy of Eye | Intro to data collection tools – |
| 1:00 | WHO Simplified Grading | Household survey and census |
|  |  |
|  |  |  |



|  | Practice Slides | Discuss question translation |
| --- | --- | --- |
|  |  |  |
| 1:00 – | Lunch | Lunch |
| 1:45 |  |  |
|  |  |  |
| 1:45 – | Examination Techniques | Role play (Demonstration (or |
| 3:45 | Practice Slides | consent/assent) and discussion then |
|  |  | break into pairs for each person to |
|  |  |  |
|  | Concretions | conduct 2 full interviews) |
|  |  |  |
|  | Practice Slides |  |
|  |  |  |
| 3:45 – | Tea | Tea |
| 4:00 |  |  |
|  |  |  |
| 4:00 – | Reliability exam – slides | Continue role play exercises |
| 5:30 |  |  |
|  |  |  |

**Wednesday 6 June**

8:30 Assembly

| **Time** | **1. Trachoma Graders – Lisa** |  | **2. Recorder – Lisa** |
| --- | --- | --- | --- |
|  |  |  |  |
| 8:30 – | Introduction to Tablets |  |  |
| 1:00 | Describe hardware, battery chargers, case | |  |
|  |  |
|  | Care / handling, on/off, home screen, buttons | |  |
|  |  | |  |
| 10:00– | Tea | |  |
| 10:15 |  |  |  |
|  |  | |  |
| 10:15— | Power management | |  |
| 1:00 | Describe GPS | |  |
|  |  |
|  |  | |  |
| 1:00 – | Lunch | |  |
|  | Common user errors | | |
|  |  | | |
| 3:45 – | Tea | | |
| 4:00 |  | | |
|  |  | | |
| 4:00 – | Role play: practice using the survey with tablets | | |
| 5:30 | (each person enters 4 HH surveys in two clusters) | | |
|  |
|  |  | | |

| **Thursday 7 June** | |  |
| --- | --- | --- |
| 7:00 AM | Assembly – discuss field exercises |  |
|  |  |  |
| **Time** | **1. Trachoma Graders – Pateh** | **2. Recorders Lisa** |
|  | **Makalo** |  |
|  |  |  |
| 9:00- | Field exercise: trachoma grading in | Field exercise: mapping, segmenting, |
| 12:30 | school children in Langtang South | practice HH surveys and census |
|  | (in HH if school is not accessible) |  |
|  |  | (1 Tablet per team. Recorders fill both |
|  |  | paper and electronic survey alternating |
|  |  | at each household) |
|  |  |  |


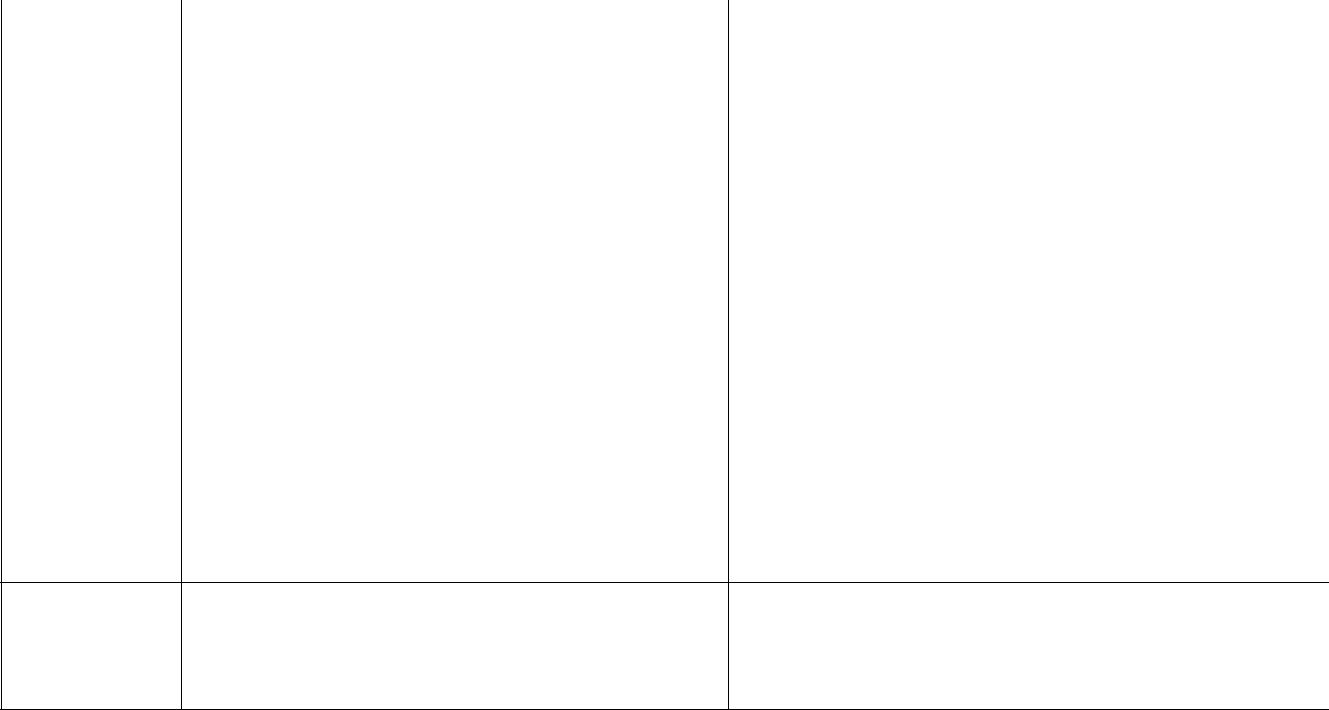


| 1:00 – | Lunch | Lunch |
| --- | --- | --- |
| 1:45 |  |  |


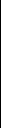


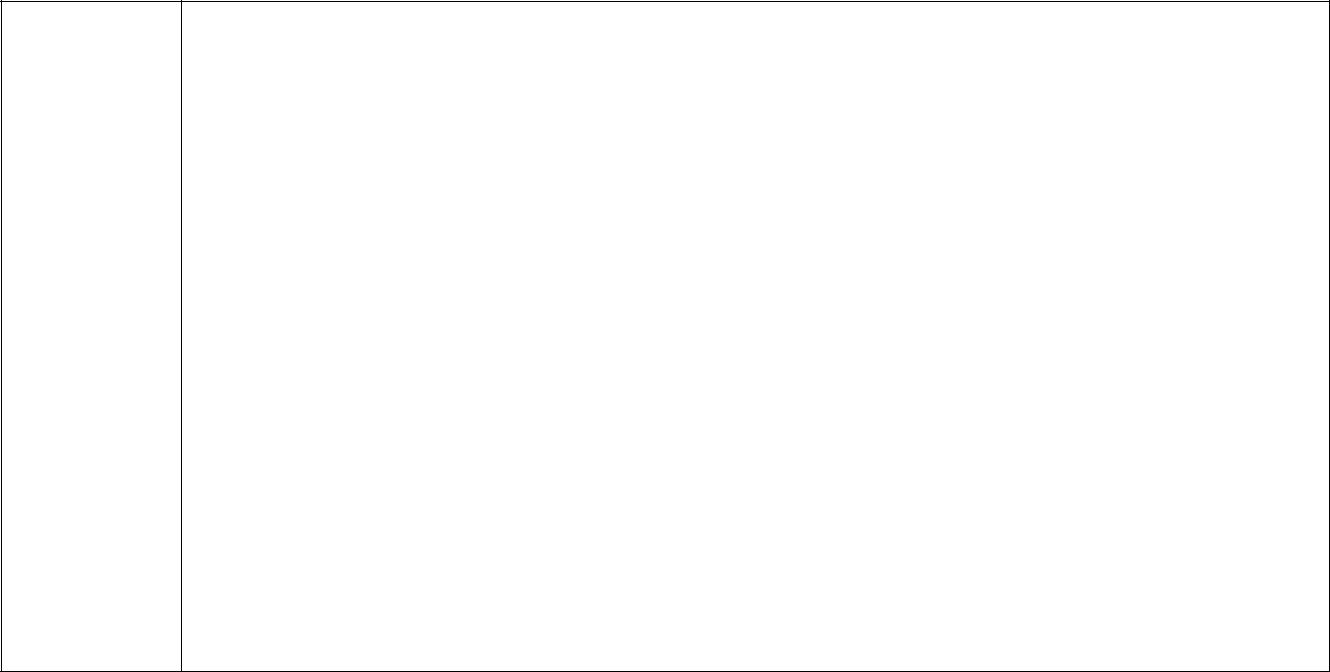


1:45-5:30 Field exercise

(1 Tablet per team. Recorders fill both paper and electronic survey alternating at each household)

Each team does 8 households

Discussion of today’s field exercise.

Any difficulties or operational issues?

**Friday 8 June, 2012**

| 7:00AM | Graders assemble |  |  |
| --- | --- | --- | --- |
| 8:30AM | Recorders assemble |  |  |
|  |  |  |  |
| **Time** | **1. Trachoma Graders – Pateh** | **2. Recorders Lisa/Nimzing** | |
|  | **Makalo** |  |  |
|  |  |  |  |
| 8:30- | Field exercise: | Discussion of field exercises | |
| 10:00 | Practice grading at school Kanam | How to remain connected to the | |
|  |
|  | (in HH if school is not accessible) | interview respondent | |
|  |  |  |  |
| 10:00- |  | Tea |  |
| 10:15 |  |  |  |
|  |  |  | |
| 10:30- |  | More role play in pairs, each person | |
| 1:00 |  | completes 5 HH survey forms and | |
|  |  | census forms | |
|  |  |  | |
| 1:00-1:45 | *Lunch* | Lunch | |
|  |  |  |  |



| 1:45 – | Field exercise: examining | Skills Exam – |
| --- | --- | --- |
| 4:00 | household members as will be | An interview will take place; each |
|  | done in actual survey |  |
|  |  | recorder must complete the survey |
|  |  |  |
|  |  | (entered data will be reviewed for |
|  | Discuss field exercise | correctness and completeness) |
|  |  |  |
|  |  |  |
| 4:00 – |  | Tea |
| 4:15 |  |  |
|  |  |  |
| 4:15 – |  | Discussion of skills exam |
| 5:30 |  |  |

**Saturday 9 June, 2012** (open for additional training and organization of survey teams):

| **Time** | **1. Trachoma Graders – Pateh** | **2. Interviewers / Recorders** |
| --- | --- | --- |
|  | **Makalo** |  |
|  |  |  |
| 8:30- | Discussion of field experience |  |
| 10:00 | Selection of teams |  |
|  |  |
|  | Distribution of materials |  |
|  |  |  |
| 10:00- | Tea |  |
| 10:15 |  |  |
|  |  |  |
| 10:15- |  |  |
| 1:00 |  |  |
|  |  |  |
| 1:00-1:45 | Lunch |  |
|  |  |  |
| 1:45 – | Open for discussion |  |
| 3:45 |  |  |
|  |  |  |
| 3;45 – | Tea |  |
|  |  |  |



4:00


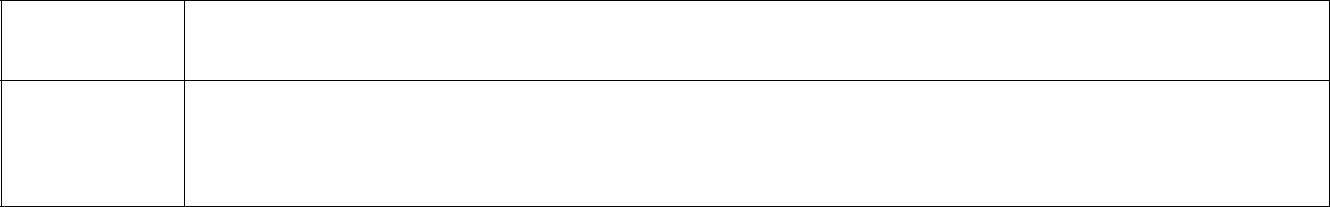


4:00 – Open for discussion

5:30

**Sunday 3 June**

3:00 PM Organization and deployment of survey team



| **Time** | **1. Trachoma Graders – Pateh** | **2. Recorders Lisa** |
| --- | --- | --- |
|  | **Makalo** |  |
|  |  |  |
| 9:00- | Field exercise: trachoma grading in | Field exercise: mapping, segmenting, |
| 12:30 | school children in Langtang South | practice HH surveys and census |
|  | (in HH if school is not accessible) |  |
|  |  | (1 Tablet per team. Recorders fill both |
|  |  | paper and electronic survey alternating |
|  |  | at each household) |
|  |  |  |
| 1:00 – | Lunch | Lunch |
| 1:45 |  |  |
|  |  |  |
| 1:45-5:30 | Field exercise |  |
|  | (1 Tablet per team. Recorders fill both paper and electronic survey | |
|  | alternating at each household) |  |
|  | Each team does 8 households |  |
|  | Discussion of today’s field exercise. |  |
|  | Any difficulties or operational issues? | |
